# Regulation of O-GlcNAcylation on endothelial nitric oxide synthase by glucose deprivation and identification of its O-GlcNAcylation sites

**DOI:** 10.1038/s41598-020-76340-7

**Published:** 2020-11-09

**Authors:** An He, Shupeng Hu, Qiangzhong Pi, Yongzheng Guo, Yang Long, Suxin Luo, Yong Xia

**Affiliations:** 1 Division of Cardiology, The First Affiliated Hospital of Chongqing Medical University, Chongqing, 400016 China; 2 Institute of Life Science, Chongqing Medical University, Chongqing, 400016 China

**Keywords:** Metabolomics, Cardiovascular biology, Biochemistry, Cardiology

## Abstract

As an energy-sensitive post-translational modification, O-GlcNAcylation plays a major role in endothelial nitric oxide synthase (eNOS) activity regulation. However, effects of glucose deprivation on eNOS O-GlcNAcylation and the presence of novel O-GlcNAcylation sites of eNOS under glucose deprivation remain unknown. Hence, we aim to determine the effects of glucose deprivation on O-GlcNAcylation and novel O-GlcNAcylation sites of eNOS. Bovine aortic endothelial cells (BAECs) and Sprague–Dawley rats were induced by glucose deprivation and their eNOS O-GlcNAcylation was subjected to immunoblotting. eNOS and transfected eNOS were purified by pull-down assay and immunoprecipitation respectively. Novel O-GlcNAcylation sites of eNOS were predicted by HPLC–MS and MS/MS Ion and determined by immunoblotting. eNOS activity was detected by Elisa and isotope labeling method. In BAECs and rat thoracic aorta, low glucose-associated activation of eNOS was accompanied by elevated O-GlcNAcylation, which did not affect O*-*linked serine phosphorylation at 1179/1177 residues. Changes in this post-translational modification were associated with increased O-GlcNAc transferase (OGT) expression and were reversed by AMPK knockdown. Immunoblot analysis of cells expressing His-tagged wild-type human eNOS and human eNOS carrying a mutation at the Ser1177 phosphorylation site confirmed an increase in O-GlcNAcylation by glucose deprivation. A marked increase in O-GlcNAcylation indicated that eNOS contained novel O-GlcNAcylation sites that were activated by glucose deprivation. Immunoblot analysis of cells expressing His-tagged human eNOS carrying a mutation at Ser738 and Ser867 confirmed an increase in O-GlcNAcylation by glucose deprivation. Conversely, in His-tagged human eNOS carrying a mutation at Thr866, O-GlcNAcylation was unaffected by glucose deprivation. Differences in culture conditions were identified using two-way analysis of variance (ANOVA), one-way ANOVA, and unpaired Student’s *t*-test. Glucose deprivation increases O-GlcNAcylation and activity of eNOS, potentially by the AMPK-OGT pathway, suggesting that Thr866 is a novel O-GlcNAcylation site involved in glucose-deprivation mediated eNOS activation.

## Introduction

eNOS plays an important role in regulating the cardiovascular system. It also functions in the synthesis of endogenous vasodilator nitric oxide (NO), which is essential for vasodilatation^1^. Endothelial NO also contributes to vascular homeostasis by regulating cell growth, platelet aggregation, and leukocyte binding to endothelial cells^2–4^. Endothelium-dependent relaxation is dysregulated in both micro-circulation and macro-circulation during acute hypoglycemia in normal and diabetic patients, suggesting that eNOS activity may be affected by hypoglycemia^5,6^. eNOS activity is subject to several overlapping modes of post-translational modifications. Phosphorylation and O-GlcNAcylation are two principal post-translational modifications of eNOS that provide mechanisms for dynamic stimulation and inhibition of enzyme activity^7^.

As a nutrient-sensitive post-translational modification, O-GlcNAcylation controls the intensity of signal traveling through different pathways based on the trophic state of cells^8^. However, the major pathway controlling the O-GlcNAcylation of eNOS and underlying mechanisms by which eNOS activity is altered by hypoglycemia remains unknown. The major biochemical pathway controlling hypoglycemia-induced O-GlcNAcylation has been discovered recently indicating AMPK activation was induced by hypoglycemia^9^. Hypoglycemia-induced activation of OGT via AMPK and the OGT activation increased protein O-GlcNAcylation^9,10^. We therefore hypothesized that hypoglycemia might affect eNOS activity via AMPK-induced OGT activation, resulting in O-GlcNAcylation and changes in NO production.

O-GlcNAcylation frequently competes with phosphorylation in cellular regulatory pathways, and competition predictive servers have identified several Ser and Thr residues in eNOS as possible targets of either phosphorylation or O-GlcNAcylation^11,12^. In eNOS, hyperglycemia increases the level of O-GlcNAcylation, and reciprocally decreases the phosphorylation level of Ser-1177^12,13^. Previous investigations have focused on how hyperglycemia induces competitive modification imbalance between O-GlcNAcylation and phosphorylation in eNOS; however, few studies have analyzed effects of hypoglycemia on this competitive modification^14^. This study aims to elucidate the glycosylation of eNOS sites in response to hypoglycemia and to identify potential sites of O-GlcNAcylation. To identify the potential O-GlcNAcylation sites, glycosylated amino acid residues were identified by fractionation of chymotryptic peptides using high performance liquid chromatography-mass spectrometry (HPLC–MS).

## Methods

### Plasmid construction

eNOS-wild-type (WT)—The cDNA sequence of human eNOS (kindly provided by Dr. Yong Xia) was subcloned into pCDN3.1 (His-tagged). eNOS-S1177A, eNOS-T866A, eNOS-S867A, and eNOS-S738A were point mutants of eNOS-WT constructed by site-directed mutagenesis (Ser1177, Thr866, Ser867, and Ser738 mutated to Ala). All mutations were performed by TransGen (TransGen Biotech, Beijing, China) and were confirmed by direct sequencing.

### Cell culture, transfection, and glucose deprivation induction

BAECs (6–10 passages) and human embryonic renal (HEK293) cells were cultured in DMEM (Invitrogen, Carlsbad, CA, USA) supplemented with 10% (v/v) FBS (Invitrogen) and 1% (v/v) penicillin/streptomycin (PS) (Beyotime Biotechnology, Jiangsu, China) in a humidified incubator of 5% CO_2_ at 37 °C. For cell transfection, BAECs cultured in a 100-mm dish were transfected with plasmid encoding eNOS-WT and eNOS mutants using Lipofectamine 3000 (Invitrogen) following the manufacturer’s instructions of use and analyzed 48 h after transfection. HEK293 cells in 6-well cell culture plates were transfected with plasmid encoding eNOS-WT or eNOS mutants using Lipofectamine 2000 (Invitrogen) following the manufacturer’s instructions of use and analyzed 24 h after transfection. To induce low glucose, BAECs and HEK293 cells were cultured in the medium containing 0, 0.5, 1, and 2.5 mmol/L glucose, respectively. Cells cultured in 5.5 mmol/L glucose medium were served as control.

### AMPKα1 gene silencing in BAECs

The sense (5′-GAUCCAUCAUAUAGCUCAAdTdT-3′) and antisense (3′-UUGAGCUAUAUGAUGGAUCdTdT-5′) small interfering RNA (siRNA) strands of *AMPKα1* were purchased from Biomic (Nanjing, China). siRNA oligonucleotides (75 nM) were delivered into cells using Opti-MEM (Invitrogen) and Lipofectamine 2000 according to the manufacturer’s protocol. Forty-eight hours after transfection, BAECs were divided into the following six groups: the control, low glucose (1 mM), low glucose (1 mM) + AMPK negative control (NC), low glucose (1 mM) + AMPK siRNA, low glucose (1 mM) + AMPK siRNA + AICAR (500 μM), and the AICAR-treated group. AICAR acted as an activator of AMPK.

### Preparation of protein samples from cells

After culturing, cells were harvested by scraping. The collected cells were washed three times with ice-cold PBS, resuspended in 100 μL lysis buffer (Beyotime) containing protease inhibitor cocktail (Beyotime) and PUGNAc (Sigma Aldrich, St. Louis, MO, USA) in a clean Eppendorf tube, and kept on ice for 1 h. The homogenate was then centrifuged at 13,800 × *g* and 4 °C for 15 min and the supernatant was recovered. The concentration of the supernatant was determined by the Bradford protein assay.

### Preparation of protein samples from rat aortae

Male and Female Sprague–Dawley rats aged approximately 6 to 8 weeks, weighing 180 to 220 g, were purchased from the Experimental Animal Center of Chongqing Medical University. All animal procedures were carried out according to the guidelines of the China Animal Protection Law and were approved by the Institutional Ethics Committee of Chongqing Medical University [Permit No. SCXK (Chongqing) 2007–0001] and the State Science and Technology Commission of China. The animals were housed individually and exposed to a cycle of 12 h of light and 12 h of darkness at 22–23 °C, with ad libitum access to food and water.

After overnight fasted, rats (200–250 g) were randomly divided into four groups (5 in each group): the control group, groups of hypoglycemia for 3, 6, and 9 h respectively. In hypoglycemia groups, rats received glargine insulin (Lantus-Solostar, Paris, France) subcutaneous injection (200 g/U). The control group was subcutaneously injected with PBS (200 g/1 ml). After insulin injection, blood glucose was monitored via tail prick every 30 min from 0 to 9 h, Hypoglycemia was confirmed when the glycemic index was less than 3 mmol/L^14,15^. Rats were anesthetized with subcutaneous injection (0.002 ml/g) of a 2% sodium pentobarbital solution. The thoracic aorta was quickly removed and washed with ice-cold PBS before the rat thoracic aortae were cut into pieces. Tissues were resuspended in a lysis buffer containing protease inhibitor cocktail and PUGNAc in a clean Eppendorf tube. Tubes were placed on a cracker and the tissues were homogenized at 4 °C for 70 min. Lysate supernatants were collected by centrifugation at 13,800 × *g* 4 °C for 15 min. The concentration of the supernatant was determined by the Bradford protein assay.

#### Vascular reactivity in isolated aortic arteries

The thoracic aorta of the control, hypoglycemia, and AMPK inhibitors rats were immediately exposed the 0–4 °C Kerbs solution filled with 95% O_2_ and 5% CO_2_. In AMPK inhibitors group, Compound C (0.2 mg/kg) was administrated 30 min before an insulin injection. The aorta was cut into four rings of 3–4 mm in length and placed in a 37 °C water bath containing 8 ml Kerbs solution. Changes in vascular tension were continuously recorded by BUXCO biological recording system, and 95% O_2_/5% CO_2_ was continuously input: Kerbs solution was refreshed every 15 min. Aortic rings were given an optimal resting tension of 2.0 g and balanced for 60 min. Before the experiment, rings were contracted with 60 mM KCl to assess their contractility. Contractile responses were assessed by the incubation of aortic segments with phenylephrine (Phe, 10^−8^ to 10^−6^ M). Endothelial integrity was analyzed by adding acetylcholine (Ach, 10^−8^ to 10^−4.5^ M) to segments pre-contracted with Phe (10^−7^ to 10^−6^ M to achieve an equivalent tone between groups). The average tension of the four aorta rings was recorded at each concentration of Ach. Endothelium-dependent vasodilatation response (EDVR) = ((maximum tension − tension at each concentration point)/(maximum tension − base value)) × 100%.

### Immunoprecipitation

For immunoprecipitation, aliquots of cell homogenates were incubated with polyclonal antibodies targeting OGT (4 μg/ml) (Abcam, Cambridge, UK) at 4 °C overnight. Protein G-Sepharose beads (GE Healthcare, Chicago, IL, USA) were added to the supernatant and incubated for 3 h. The beads were washed thoroughly with lysis buffer and then eluted in SDS-PAGE sample buffer (Beyotime) by boiling for 3 min. Proteins were revealed by western blot.

### eNOS pull-down assay

In BAEC and rat thoracic aorta, eNOS was extracted from the lysate by affinity precipitation using 2′,5′-ADP Sepharose beads (GE Healthcare), as described previously^12^. Cell/tissue lysates were mixed with the prepared 2′,5′-ADP Sepharose resin (50% slurry) at 4 °C for 2 h and gently shaken. The mixture was then centrifuged at 13,800 × *g* for 1 min. The supernatant was discarded, and the resins were washed with washing buffer (PBS supplemented with 500 mM NaCl) three times. Bound proteins were eluted by boiling the resins in 50 μL SDS-PAGE sample buffer for 10 min. For transfected cells, eNOS was extracted by affinity precipitation after transfection using the His-tag protein purification Kit (Beyotime) as per the manufacturer’s protocol. Total eNOS after affinity purification was used for subsequent immunoblotting assay.

### Immunoblotting

For western blot, proteins were fractionated by SDS-PAGE and transferred to PVDF membranes (Bio-Rad, Hercules, CA, USA). After blocking in the buffer (20 mM Tris–HCl (pH 7.5), 150 mM NaCl, 5% skim milk powder, 0.1% (v/v) Tween-20) at room temperature for 1.5 h, the membrane reacted with appropriate primary antibodies (primary antibodies 1:2000; diluted in blocking buffer) at 4 °C overnight. Thereafter, membranes were thoroughly washed with TBST (20 mM Tris–Cl (pH 7.5), 150 mM NaCl, 0.1% (v/v) Tween-20) three times, each time for 10 min. Membranes were then incubated in 1:5000 diluted secondary antibody for 1.5 h, thoroughly washed with TBST three times (10 min per wash), and finally detected by chemiluminescence (Advansta Inc., San Jose, CA, USA). Results were quantified by densitometry using Image J 1.8.0 software (National Institutes of Health).

Primary antibodies targeting phospho-eNOS Ser1177, phospho-eNOS Ser633, phospho-eNOS Thr495, and eNOS were purchased from Cell Signaling Technology (Danvers, MA, USA); O-GlcNAc antibody (RL2) was purchased from Thermo Fisher Scientific (Waltham, MA, USA). Antibodies targeting O-GlcNAcase (OGA), Threonine, OGT, phospho-AMPK Thr172, and anti-AMPKα1 were purchased from Abcam (Cambridge, UK); β-actin antibody was purchased from Proteintech (Rosemont, IL, USA). Goat anti-mouse IgG and goat anti-rabbit IgG were purchased from Santa Cruz Biotechnology (Dallas, TX, USA) and Proteintech, respectively. Membranes used for O-GlcNAc analysis were stripped and probed with rabbit polyclonal anti-P-eNOS (Ser-1177) Ab (Cell Signaling Technology), and then stripped and reprobed for total eNOS. Probed at 4 °C with anti-phospho-OGT-thr antibody, and then stripped and reprobed for total OGT.

### Preparation of eNOS for HPLC–MS

eNOS was extracted from eNOS-WT-transfected HEK293 cells by affinity precipitation using the His-tag protein purification kit. The day before harvest, cells were incubated with low glucose at 1 mM for 6 h. The affinity-precipitated eNOS was fractionated by SDS-PAGE, and the gel was stained with Coomassie brilliant blue. Stained eNOS was excised from the gel and stored in a clean Eppendorf tube after the excised gel was confirmed the inclusion of eNOS by immunoblotting using the Micro Protein PAGE Recovery Kit (Sangon Biotechnology, Shanghai, China). The gel in the Eppendorf tubes was decolorized and swelled. After de-coloration and swelling, absolute acetonitrile was added to shrink and solidify the gel. Finally, the acetonitrile was absorbed and the gel was thermally dried. Dithiothreitol (DTT) solution was added to the tubes, mixed thoroughly, and thermally incubated at 56 °C for 1 h; after incubation, the solution was discarded and the sample was thermally dried. Indole-3-acetic acid (IAA) solution was added, mixed well, and the mixture was incubated at room temperature for 30 min; thereafter, the solution was discarded and DTT was added. The mixture was incubated at room temperature for 15 min to neutralize the remaining IAA; the solution was then discarded and the sample was thermally dried. Chymotrypsin was added to the sample at an enzyme/protein ratio of 1:20 and incubated at 37 °C for 14 h in a reaction system containing 200 μL 50 mmol/L ammonium bicarbonate solution. After the enzymatic digestion was completed, 1% TFA solution, 60% acetonitrile solution, 0.1% TFA solution, and absolute acetonitrile solution were successively added into the enzymatic digestion solution. The reaction was conducted at 37 °C for 1 h respectively, and the reaction solution was combined.

### HPLC–MS for enrichment of eNOS O-glycosylation

Digested samples were dissolved in A solution (deionized H_2_O containing 0.1% formic acid) and centrifuged at 1000 × *g* for 5 min; the supernatants were then tested using the Thermo Orbitrap Lumos HPLC–MS system with a data collection time of 120 min, spray voltage of 2.20 kV, the capillary temperature of 320 °C, collision energy of 50%, a first-level mass range of collection of 300–1800 m/z. Data dependent analysis selected the ten most highly abundant ions for subsequent MS/MS analysis. And second-level scanning range of 100–1400 m/z. Data on eNOS O-glycosylation enrichment by HPLC–MS were provided by the Beijing Proteome Research Center Tandem Mass Spectrometry (MS/MS) Laboratory (China). Using the Mascot software to search MS/MS spectra in the mammalian’ NCBI database, increased 204 Dalton’ serine/threonine was identified as a potential modification site of HexNAc glycosylation.

### Measurement of NO

The concentration of NO released from BAECs in culture medium was measured by the concentration of nitrate and nitrite using a modified Griess reaction method with the Total Nitric Oxide Assay Kit (Beyotime). Aorta blood samples from normal and hypoglycemic rats were collected using vacuum hemostix. Plasma was stored at − 80 °C for assay. NO concentrations were determined using commercially available enzyme-linked immunosorbent assay (ELISA) kits (Nanjing Jiancheng Bioengineering Institute, Nanjing, China) as per the manufacturer’s protocol.

### eNOS activity assay

eNOS activity was determined by converting rate of L-[14C] arginine to L-[14C] citrulline in cells. The conversion was monitored in a 300 μL buffer containing 50 mM Tris–HCl, pH 7.4, 5 μM L-[14C] arginine (Moravek, Brea, CA, USA), 45 μM L-arginine, 0.5 mM NADPH, 10 μM BH4, 10 μg/ml calmodulin, and 10 nM eNOS. The reactions were initiated by adding L-[14C] arginine and terminated in a stop buffer (20 mM HEPES, 2 mM EDTA) after incubation at 37 °C for 1 h. L-[14C] citrulline was separated from the reaction mixture by Dowex AG 50W-X8 (Na^+^ form) (Sigma) cation exchange columns and quantitated by liquid scintillation counting. N(gamma)-nitro-L-arginine methyl ester (L-NAME; 5 mM) inhibitory activity was analyzed to determine the concentration of L-[14C] citrulline converted by eNOS.

### Statistical analysis

All data were presented as the mean ± standard error of the mean (SEM). Statistical analysis was performed using SPSS by two-way analysis of variance (ANOVA), one-way ANOVA, or unpaired Student’s *t*-test. A value of *P* less than 0.05 was considered statistically significant. Data analysis was performed using SPSS version 25 (SPSS Inc/IBM, Chicago, Ill, USA).

### Ethics approval and consent to participate

All animal procedures were carried out according to the guidelines of the China Animal Protection Law and were approved by the Institutional Ethics Committee of Chongqing Medical University [Permit No. SCXK (Chongqing) 2007–0001] and the State Science and Technology Commission of China.

## Results

### Glucose deprivation increases eNOS O-GlcNAcylation and has no effect on O-linked phosphoserine (Ser1179)

**Figure 1:**
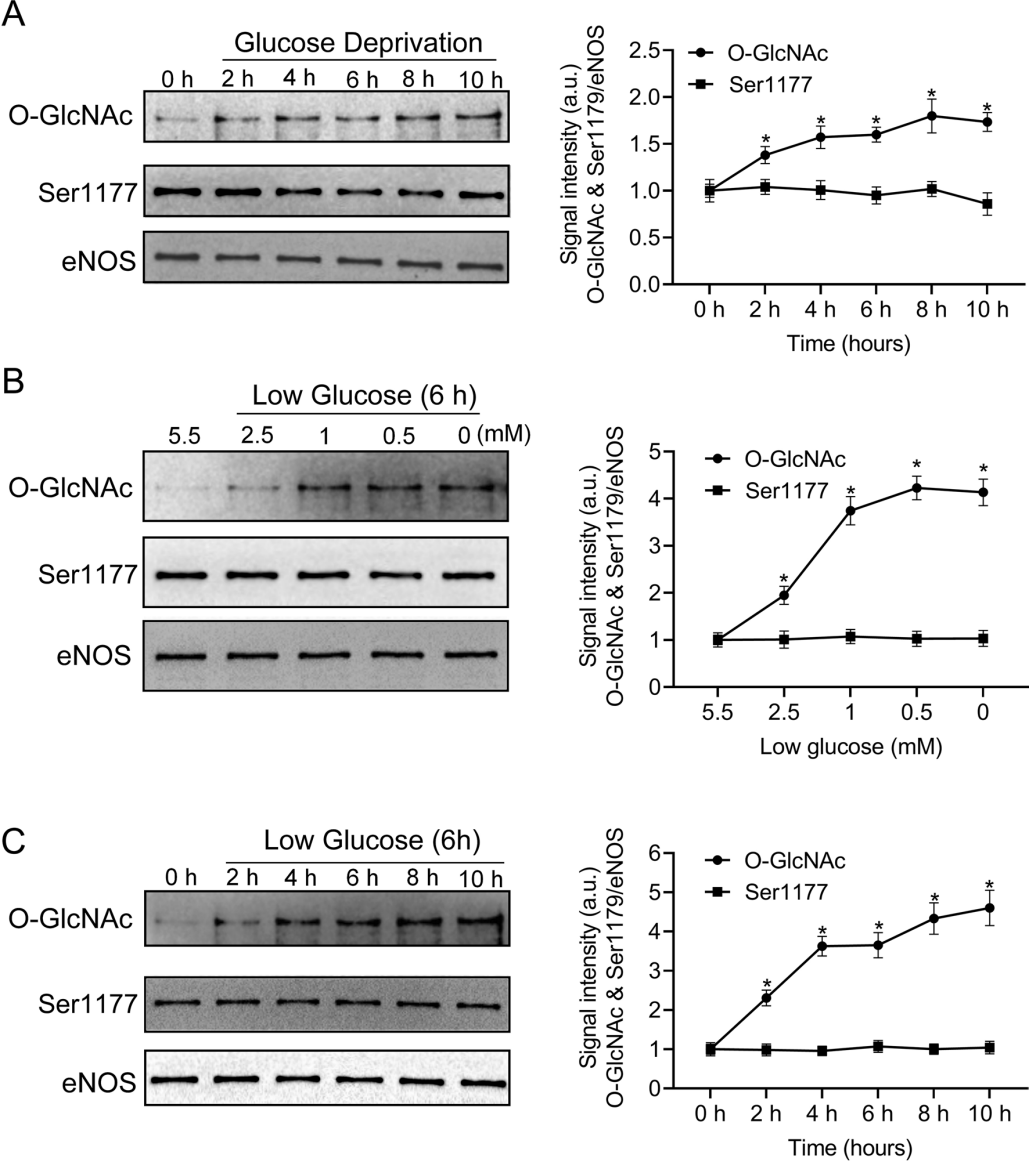
Effect of glucose deprivation on bovine aortic endothelial cell (BAEC) endothelial nitric oxide synthase (eNOS) O-GlcNAcylation. (**A**) Left panel: representative western blot of BAEC eNOS O-GlcNAcylation and phospho-eNOS (Ser1177) from control or glucose deprivation-treated cells (n = 3) over 10 h. Right panel: quantification of the ratio between O-GlcNAcylation, phosphorylated eNOS (P-eNOS), and eNOS. (**B**) Left panel: representative western blot of BAEC O-GlcNAcylation, P-eNOS, and eNOS in response to a decrease in extracellular glucose from 5.5 to 0 mM (n = 3) over 6 h. Right panel: quantification of the ratio between O-GlcNAcylation, P-eNOS, and eNOS. (**C**) Left panel: representative western blot of BAEC eNOS O-GlcNAcylation and phospho-eNOS (Ser1177) from control or 1 mM glucose-treated cells (n = 3) over 10 h. Right panel: quantification of the ratio between O-GlcNAcylation, P-eNOS, and eNOS. Full-length blots are presented in Supplementary Figure S1 (A), S2 (B), and S3 (C). Data are shown as the mean ± SEM. Signal density of O-GlcNAc and P-eNOS (Ser-1177) bands relative to the corresponding eNOS bands. Values are normalized to control (0 h). * *P* < 0.05 vs. control. Squares = O-GlcNAc; circles = P-eNOS (Ser 1179).

To confirm that glucose deprivation affects eNOS O-GlcNAcylation, western blot was performed to detect O-GlcNAcylation and phospho-eNOS (Ser1179) under glucose deprivation. As demonstrated in Fig. 1A, the ratio of eNOS O-GlcNAcylation to eNOS increased by 1.7 folds after 10 h of glucose deprivation, whereas phospho-eNOS (Ser1179) was not affected, suggesting that glucose deprivation only elevated eNOS O-GlcNAcylation. To determine the concentration of glucose with the same effect on eNOS as glucose deprivation, western blot was performed to detect O-GlcNAcylation and phospho-eNOS (Ser1179) at different concentrations of low glucose. As demonstrated in Fig. 1B, the ratio of eNOS O-GlcNAcylation to eNOS increased by 3.7 folds after 6 h of graded glucose concentration reduction from 5.5 to 1 mM; this ratio continued to increase from 3.7 to 4.1 folds following a graded reduction from 1 to 0 mM. In contrast, phospho-eNOS (Ser1179) did not change, suggesting that the effect of 1 mM glucose on eNOS O-GlcNAcylation was identical to glucose deprivation. As demonstrated in Fig. 1C, 1 mM glucose produced the same effect as glucose deprivation. Moreover, total global O-GlcNAc levels were higher in glucose deprivation than in the control (supplementary material Fig. S16). These data strongly suggested that glucose deprivation only increased eNOS O-GlcNAcylation and did not affect phospho-eNOS (Ser1179).

### Low glucose increases eNOS O-GlcNAcylation through OGT up-regulation and AMPK activation

**Figure 2:**
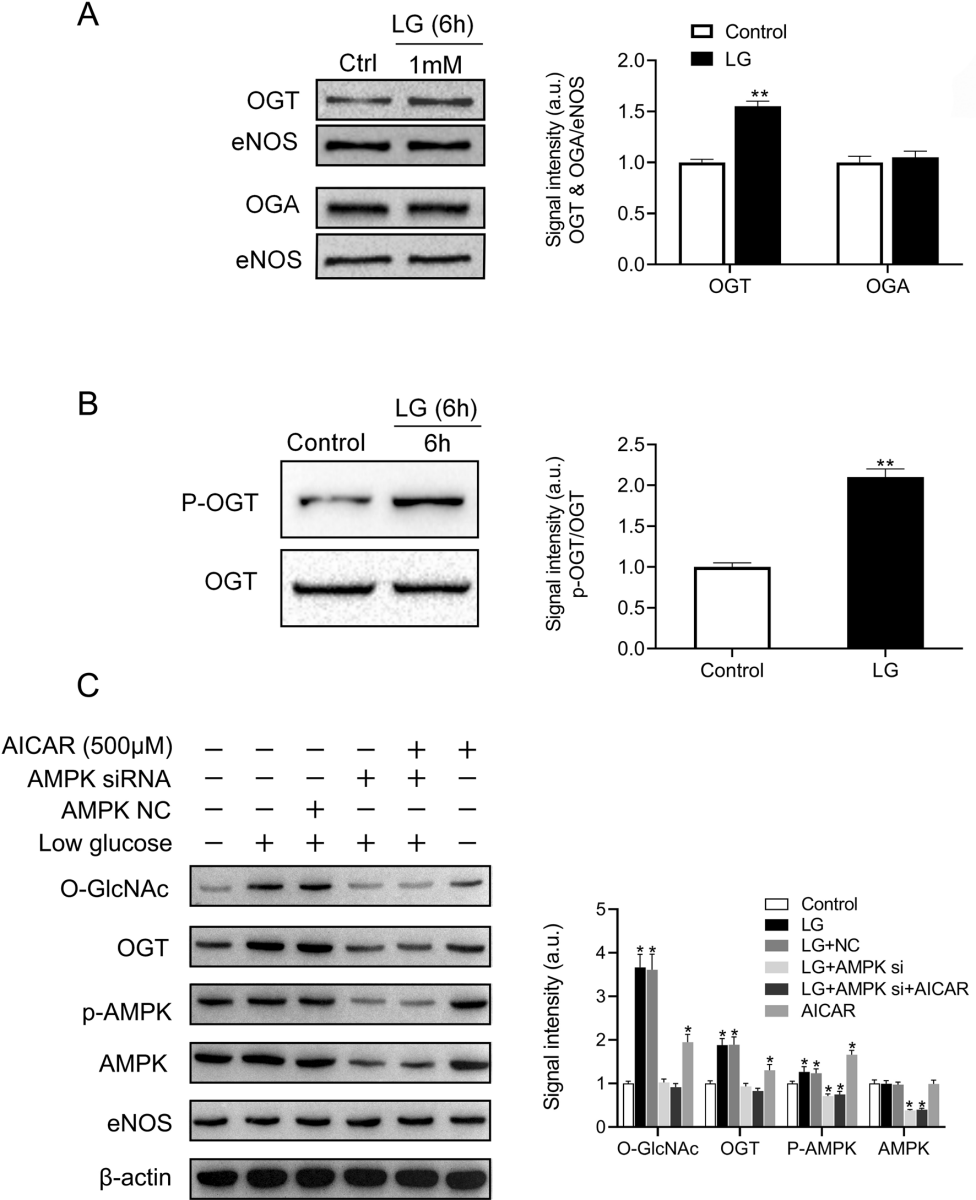
Increased O-GlcNAcylation stimulated by glucose deprivation is caused by AMP kinase (AMPK)-dependent O-GlcNAc transferase (OGT) activation. (**A**) Left panel: representative western blot of OGT and O-GlcNAcase (OGA) from control (5.5 mM) or low (1 mM) glucose (LG)-treated bovine aortic endothelial cells (BAECs) (n = 3) incubated for 6 h. Right panel: quantification of the ratio between OGT and OGA. Signal density of OGT and OGA bands relative to the corresponding eNOS bands. (**B**) Left panel: representative western blot of phospho-threonine OGT and OGT from control or LG-treated BAECs (n = 3) incubated for 6 h. Right panel: quantification of the ratio between phospho-threonine OGT and OGT. (**C**) Left panel: representative western blot of AMPK, P-AMPK, eNOS O-GlcNAcylation, and eNOS from control or LG-treated BAECs or AMPK-knockdown BAECs (n = 3) incubated for 6 h. Right panel: quantification of the ratio of AMPK, P-AMPK, eNOS O-GlcNAcylation, and eNOS. Signal density of O-GlcNAcylation and OGT bands relative to the corresponding eNOS bands. Signal density of AMPK bands relative to the corresponding β-actin bands. Signal density of p-AMPK bands relative to the corresponding AMPK bands. AICAR was an activator of AMPK. Full-length blots are presented in Supplementary Figure S4 (A), S5 (B), and S6 (C). Data are shown as the mean ± SEM, * < 0.05; ** *P* < 0.01 vs. control.

Previous investigations demonstrated that OGT and OGA were two enzymes that regulated O-GlcNAcylation cycling^16^. To identify which enzyme regulated eNOS O-GlcNAcylation under low glucose, affinity precipitation of eNOS was subjected to western blot, and antibodies targeting OGT and OGA were performed. It was found that low glucose increased OGT by 50%, while reciprocal OGA expression remained unchanged (Fig. 2A). Thr phosphorylation was noticed to activate OGT^17^; therefore, to confirm that low glucose correlated with an increase in OGT Thr-phosphorylation, the immunoprecipitation of OGT followed by western blot with antibodies targeting phospho-Thr were performed. The ratio of phospho-Thr OGT to OGT increased by 50% after incubation of 6 h under low glucose condition, suggesting that low glucose increased OGT phosphorylation at its Thr site (Fig. 2B). These data suggested that low glucose not only increased eNOS O-GlcNAcylation by inducing the binding of OGT to eNOS but also elevated OGT Thr-phosphorylation. Total OGT expression was also elevated with low glucose treatment (supplementary material Fig. S16). As a cellular energy sensor, AMPK could be activated by lowering the extracellular ATP/ADP ratio and participated in protein O-GlcNAcylation^9,17^. To confirm the effect of AMPK on eNOS O-GlcNAcylation, western blot was performed to detect O-GlcNAcylation, OGT, eNOS, P-AMPK Thr172, and AMPK. The ratio of eNOS O-GlcNAcylation and OGT to eNOS was increased, while the ratio of P-AMPK to AMPK was also increased, after 6 h of incubation under low glucose condition. However, AMPKα1 knockdown was shown to reverse these effects of eNOS O-GlcNAcylation, OGT, and AMPK phosphorylation (Fig. 2C). In addition, O-GlcNAcylation and OGT levels also increased in AICAR-treated cells, although the effect was less than those cells treated with low glucose. These data strongly suggested that low glucose increased eNOS O-GlcNAcylation and OGT in an AMPK-dependent manner.

### Hypoglycemia increases eNOS O-GlcNAcylation in rat aortae

**Figure 3:**
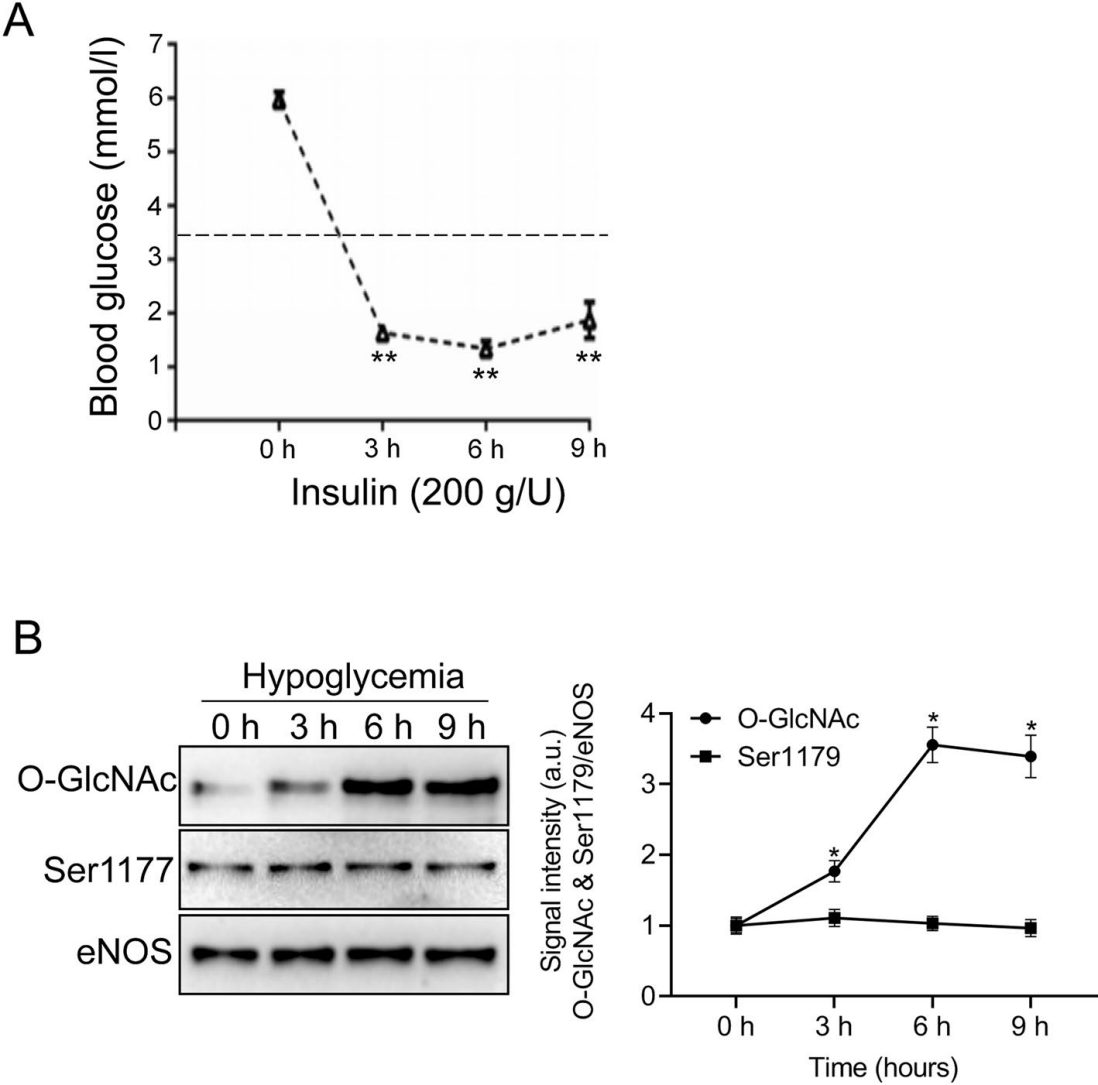
Effect of hypoglycemia on BAEC eNOS O-GlcNAcylation in rat aortae. (**A**) Blood glucose levels in response to insulin-induced hypoglycemia (200 g/U, subcutaneous) in a rat model (n = 5). (**B**) Left panel: representative western blot of O-linked Glc-NAc, P-eNOS, and eNOS from the thoracic aorta of control or insulin-treated rats injected subcutaneously with insulin (n = 5) at 3–9 h after injection. Right panel: quantification of the ratio between O-GlcNAcylation, P-eNOS, and eNOS. Data are shown as the mean ± SEM and signal density of O-GlcNAc and P-eNOS (Ser-1177) bands relative to the corresponding total eNOS bands. Full-length blots are presented in Supplementary Figure S7. ** *P* < 0.01 vs. Control (0 h).

In the above results, we identified that hypoglycemia increased eNOS O-GlcNAcylation in vitro. To confirm that hypoglycemia also increased eNOS O-GlcNAcylation in vivo, we measured the blood glucose concentration of rats after insulin injection. The blood glucose levels of rats were significantly decreased 3 h after subcutaneous insulin injection (Fig. 3A). Western blot was performed to detect O-GlcNAcylated and phospho-eNOS (Ser1177) under hypoglycemia. The ratio of eNOS O-GlcNAcylation to eNOS increased by 2.5 folds after 9 h of hypoglycemia exposure, whereas phospho-eNOS (Ser1177) was not changed (Fig. 3B). These data strongly suggested that hypoglycemia only increased eNOS O-GlcNAcylation and had no effect on phospho-eNOS (Ser1177) both in vitro and in vivo.

### Low glucose increases O-GlcNAcylation at the mutated eNOS Akt phosphorylation site

**Figure 4:**
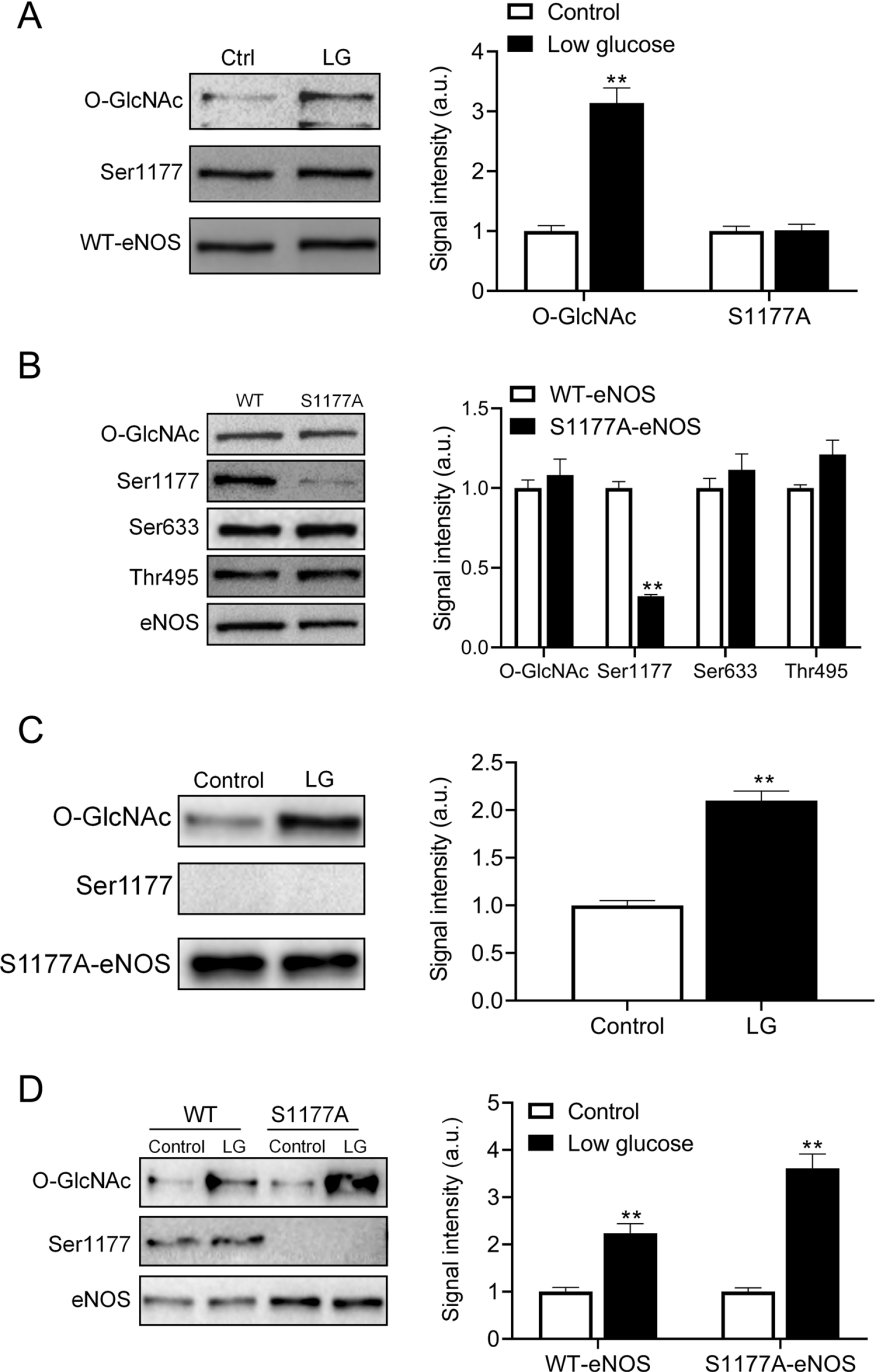
Effect of glucose deprivation on O-GlcNAcylation and phospho-eNOS (Ser1177) in His-tagged wild-type (WT)-eNOS and S1177A-eNOS. (**A**) Left panel: representative western blot of His-tagged WT-eNOS-transfected HEK293 cells, O-GlcNAcylation and phospho-eNOS (Ser1177) from control or low glucose-treated cells (n = 3) incubated for 6 h. Right panel: quantification of the ratio of O-GlcNAcylation and phospho-eNOS (Ser1177). (**B**) Left panel: representative western blot of WT-eNOS- or S1177A-eNOS-transfected HEK293 cells showing O-GlcNAcylation, phospho-eNOS (Ser1177), phospho-eNOS (Thr495), and phospho-eNOS (Ser633) under normal culture conditions. Right panel: quantification of the ratio of O-linked GlcNAc, phospho-eNOS (Ser1177), phospho-eNOS (Thr495), and phospho-eNOS (Ser633). White = WT-eNOS; black = S1177A-eNOS (**C**) Left panel: representative western blot of S1177A-eNOS-transfected HEK293 cells showing O-GlcNAcylation and phospho-eNOS (Ser1177) in control or low glucose-treated cells (n = 3) incubated for 6 h. Right panel: quantification of the ratio of O-GlcNAcylation and phospho-eNOS (Ser1177). (**D**) Left panel: representative western blot of WT-eNOS- and S1177A-eNOS-transfected BAECs showing O-GlcNAcylation and phospho-eNOS (Ser1177) in control or low glucose-treated cells (n = 3) incubated for 6 h. Right panel quantification of the ratio of O-GlcNAcylation and phospho-eNOS (Ser1177). Data are shown as the mean ± SEM and signal density of O-GlcNAc and P-eNOS bands relative to the corresponding total eNOS bands. Control, 5 Mm glucose; LG, 1 mM glucose. Full-length blots are presented in Supplementary Figure S8 (A), S9 (B), S10 (C) and S11 (D). * *P* < 0.05, ** *P* < 0.01 vs. control.

To confirm whether eNOS had a novel O-GlcNAcylation site, effects of low glucose on eNOS O-GlcNAcylation and O-linked phosphoserine at this residue were evaluated using WT-eNOS and S1177A-eNOS. As demonstrated in Fig. 4A, low glucose increased the O-GlcNAcylation of WT-eNOS and did not affect phospho-eNOS (Ser1177). As demonstrated in Fig. 4B, S1177A-eNOS still existed O-GlcNAcylation. As demonstrated in Fig. 4C, O-GlcNAcylation was increased in S1177A-eNOS in response to low glucose. As demonstrated in Fig. 4D, both BAECs transfected with WT-eNOS and S1177-eNOS increased in O-GlcNAcylation in response to low glucose. These data suggested that eNOS might have novel O-GlcNAcylation sites activated by glucose deprivation.

### HPLC–MS and MS/MS Ion search for the glucose deprivation-dependent O-GlcNAcylation sites of eNOS

**Figure 5:**
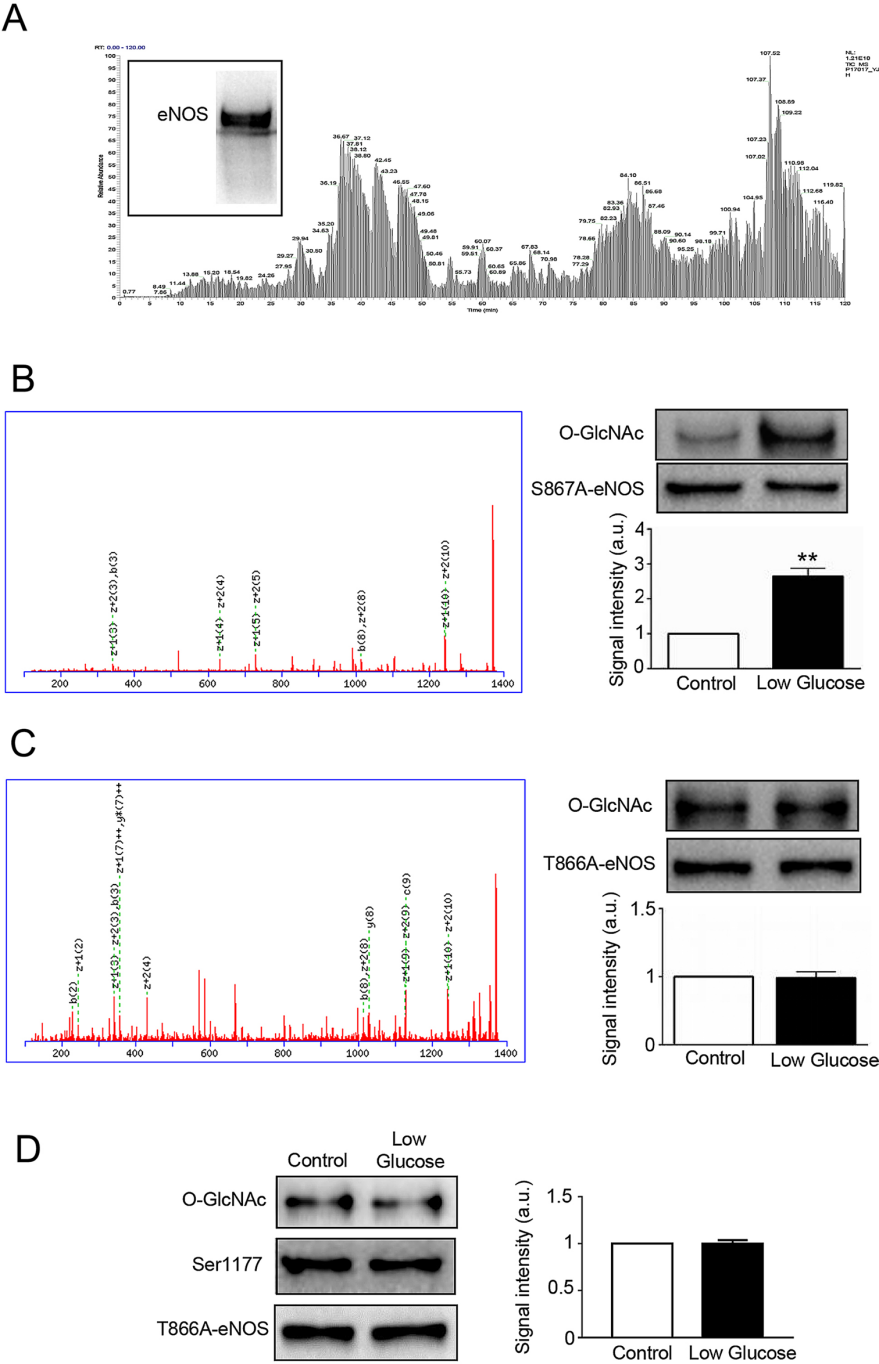
High-pressure liquid chromatography-mass spectrometry (HPLC–MS) and MS/MS Ion Search for enriched eNOS O-GlcNAcylation sites. (**A**) Inset, eNOS protein was recovered from gel. Fractionation by SDS-PAGE revealed a single major eNOS band, which was analyzed by HPLC–MS. (**B**) Left panel: MS/MS Ion Search score of eNOS Ser867 O-GlcNAcylation site. Upper right panel: representative western blot of S867A-eNOS-transfected HEK293 cells showing O-linked GlcNAc from control or low glucose -treated cells (n = 3) incubated for 6 h. Lower right panel: quantification of the ratio between control and low glucose treated O-GlcNAcylation. (**C**) Left panel: MS/MS Ion Search score of eNOS Thr866 O-GlcNAcylation site. Upper right panel: representative western blot of T866A-eNOS-transfected HEK293 cells showing O-linked GlcNAc from control or low glucose -treated cells (n = 3) incubated for 6 h. Lower right panel: quantification of the ratio between control and low glucose treated O-GlcNAcylation. (**D**) Left panel: representative western blot of T866A-eNOS-transfected BAECs showing O-GlcNAcylation from control or low glucose -treated cells (n = 3) incubated for 6 h. Right panel: quantification of the ratio between control and low glucose treated O-GlcNAcylation. Data are shown as the mean ± SEM and signal density of O-GlcNAc and P-eNOS bands relative to the corresponding total eNOS bands. Control, 5 Mm glucose; LG, 1 mM glucose. Full-length blots are presented in Supplementary Figure S12 (A), S13 (B), S14 (C) and S11 (D). ** *P* < 0.01 vs. control.

To determine whether eNOS had novel O-GlcNAcylation sites, purified eNOS samples (excised gel) were analyzed by HPLC–MS, as demonstrated in Fig. 5A. To confirm that the samples analyzed by HPLC–MS contained eNOS, proteins were recovered from the gel using Micro Protein PAGE Recovery Kit. Western blot was then performed to detect eNOS, as shown in Fig. 5A (inset). To determine whether the novel O-GlcNAcylation sites obtained by MS/MS Ion Search (Fig. 5B and 5C (left panel)) were major glucose deprivation-dependent O-GlcNAcylation sites, western blot was performed to detect O-GlcNAcylation under glucose deprivation. As demonstrated in Fig. 5B (right panel), HEK293 cells transfected with S867A-eNOS showed an increase in O-GlcNAcylation during glucose deprivation. As demonstrated in Fig. 5C (right panel), HEK293 cells transfected with T866A-eNOS showed no change in O-GlcNAcylation during glucose deprivation. Finally, as demonstrated in Fig. 5D, BAECs transfected with T866A-eNOS showed no change in O-linked GlcNAc in response to glucose deprivation. These data strongly suggested that Thr866 was a novel O-linked GlcNAc site under glucose deprivation.

### Glucose deprivation increases eNOS activity

**Figure 6:**
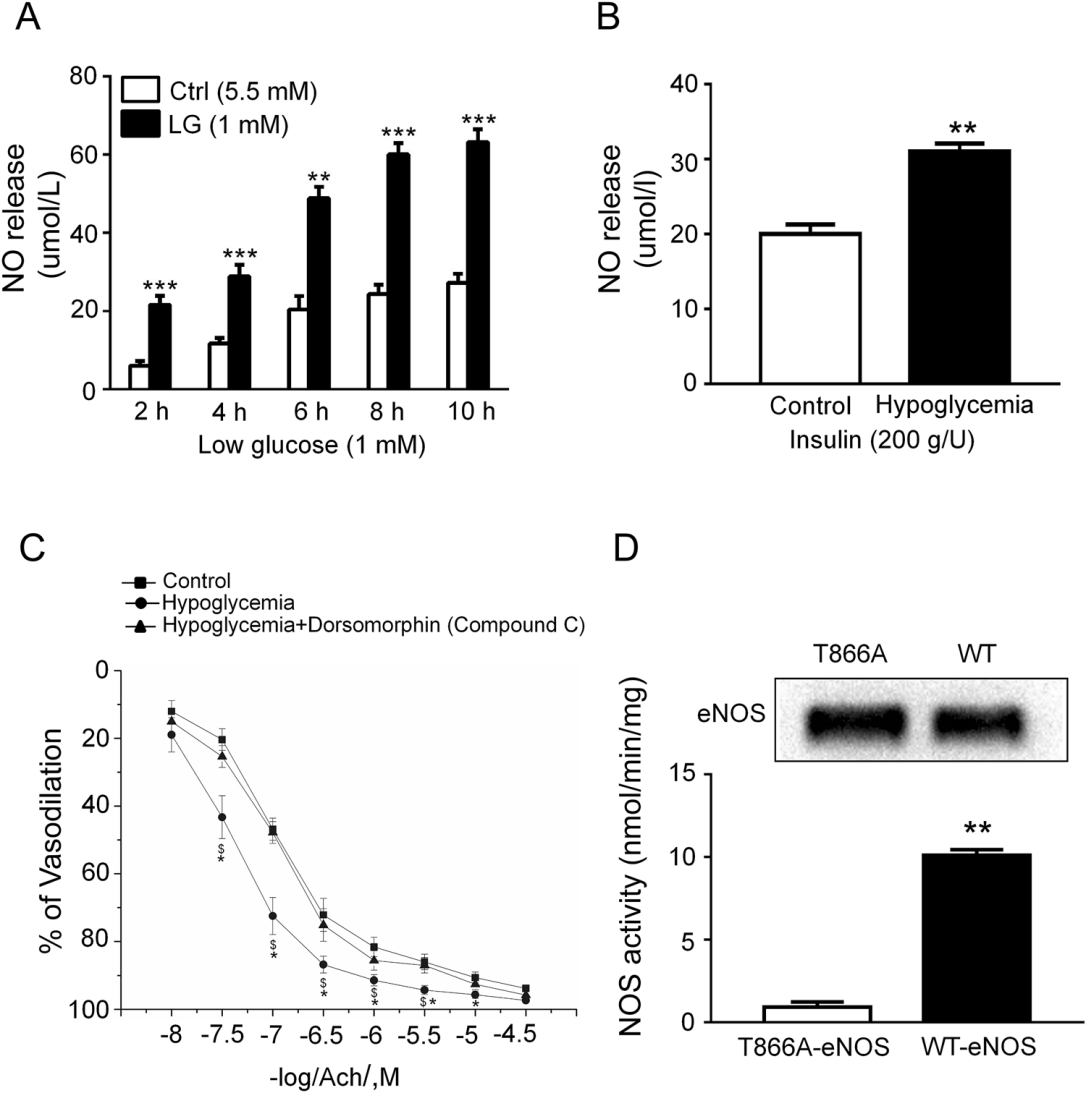
Effect of glucose deprivation on eNOS activity. (**A**) NO release in BAEC culture medium in control or low glucose-treated BAECs (n = 3) over 10 h. White = control; black = low glucose (LG). (**B**) NO release in rat aorta plasma from control or low glucose-treated rat (n = 3) after 6 h. (**C**) Cumulative concentration–response curves to acetylcholine (10^−8^–10^−4.5^ M) in aortic segments of hypoglycemia or AMPK inhibitor-treated hypoglycemia rat. Compound C (0.2 mg/kg, i.v.). Compound C was administrated 30 min before insulin (200 g/U) injection. Ach, Acetylcholine. (**D**) Effect of low glucose on L-[14C] citrulline in WT-eNOS- and T866A-eNOS-transfected HEK293 cells. Data are shown as the mean ± SEM and Control, 5 Mm glucose; LG, 1 mM glucose. Full-length blots are presented in Supplementary Figure S15. *P < 0.05, **P < 0.01, ***P < 0.001 vs. Control; $ represents hypoglycemia vs. hypoglycemia + Dorsomorphin, *P* < 0.05.

To confirm the effect of increased O-GlcNAcylation on the synthesis of NO by eNOS under glucose deprivation, we measured changes in NO production in the cell culture medium under low glucose conditions. As demonstrated in Fig. 6A, the NO production was increased by 3 folds after incubation for 10 h under low glucose conditions. Moreover, to confirm that increased O-GlcNAcylation also affected eNOS function in vivo, we measured the production of NO in rat aorta plasma. As demonstrated in Fig. 6B, the production of NO increased by 40% 6 h after hypoglycemia induction. These data strongly suggested that glucose deprivation increased the NO synthesis function of eNOS both in vitro and in vivo. Endothelial function was evaluated by analyzing effects of endothelium-dependent relaxations on Ach in aortic segments. Relaxant responses to Ach in aortic segments of the insulin-treated rats were significantly higher than those in the control group, while insulin injection after Compound C treatment showed no difference in vasodilation compared with control group (Fig. 6C). Taken together, we showed that eNOS had a novel glucose deprivation-dependent O-GlcNAcylation site. To confirm that the novel O-GlcNAcylation site would affect NO synthesis by eNOS, we analyzed changes in eNOS activity of WT-eNOS and T866A-eNOS after transfection of HEK293 cells. As demonstrated in Fig. 6C, eNOS -catalyzed L-[14C] citrulline formation by WT-eNOS was increased by 10 folds compared with T866A-eNOS (Fig. 6D). These data showed that eNOS had a novel glucose deprivation-dependent O-GlcNAcylation site and that site located at Thr866.

## Discussion

This study demonstrated that glucose-deprived BAECs increased the phosphorylation of OGT and attached to eNOS via AMPK activation, thereby inducing the eNOS O-GlcNAcylation to augment eNOS activity. When incubated in 1 mM glucose, AMPK knockdown by siRNA reversed these modifications, as expected^9,17^. Cheung et al.^9^ demonstrated that glucose deprivation induced total protein O-GlcNAcylation and OGT expression in an AMPK-dependent manner^9^. Bullen et al. (2014) reported that OGT and AMPK could directly regulate each other, AMPK phosphorylates Thr-444 on OGT, phosphorylation of Thr-444 was ultimately related to AMPK activity^17^. The function of eNOS protein was regulated by post-translational modifications including phosphorylation, glycosylation, acetylation, and palmitoylation, which affected the production of NO by altering the function and structure of eNOS^7^. These modifications probably occurred specifically at the O-GlcNAcylation site. Residues namely Ser1177 were modified by GlcNAc^12,13^; however, hypoglycemia did not reduce the rate of modification at these sites. This is the first acknowledged study to show eNOS modification by GlcNAc under hypoglycemia, and the first to reveal its underlying mechanisms. Additionally, these findings were the first example of functional alterations in a cytoplasmic enzyme induced by these modifications under hypoglycemia. Previous studies demonstrated that cell proteins could be dynamically modified by O-GlcNAc moieties due to glucose deprivation^9,10,18,19^. Although these observations were not focusing on modifications in whole cell proteins instead of endothelial cells, the same mechanism was likely to elucidate the increase in eNOS O-GlcNAcylation in glucose deprivation.

Our data were somewhat consistent with previous observations indicating that glucose deprivation increased protein O-GlcNAcylation through the up-regulation of OGT^10,17,19^. Kreppel et al.^20^ reported that UDP-GlcNAc concentrations modulates affinity of OGT for target peptides suggesting that OGT is regulated by UDP-GlcNAc levels, which is a direct substrate for the transfer of single O-GlcNAc to nuclear and cytosolic proteins by OGT^20^. Taylor et al.^10^ have discovered a significant induction of O-GlcNAc modification of a limited number of proteins under conditions of glucose deprivation. The mRNA and protein levels for nucleocytoplasmic OGT increases in glucose deprivation-treated cells compared with normal glucose-treated cells^10^. Our results confirmed increased total OGT and OGT phosphorylation in glucose deprivation-BAEC cells. We also observed an increase in O-GlcNAc modification at the whole-cell protein level in glucose deprivation treated BAECs. However, the detection of O-GlcNAcylation at the whole-cell protein level may have missed several crucial biological phenomena in response to glucose deprivation. Different post-translational modifications induced different responses to glucose deprivation, and competition was observed among those modifications^5,13^. Because we investigated O-GlcNAcylation in eNOS isolated by affinity purification, the influence of the O-GlcNAcylation of other proteins in response to glucose deprivation was excluded. The subunits in the eNOS dimer structure contain domains that bind to NADPH, and the structure of adenosine diphosphate in the 2′,5′-ADP-Sepharose is similar to NADPH, thereof eNOS can generate a strong affinity with the 2′,5′-ADP-Sepharose^21^. According to this theory, 2′,5′-ADP-Sepharose can easily and effectively detect the expression of eNOS O-GlcNAcylation. Additionally, since we identified a competitive modification site of O-GlcNAcylation by immunoblotting, these findings may contribute to the understanding of how glucose deprivation regulated eNOS activity through post-translational modifications. Low glucose induced eNOS O-GlcNAcylation and increased OGT levels, while OGA expression was unchanged. As a substrate of OGT, O-GlcNAc, a product of hexose biosynthesis pathway (HBP), participated in O-GlcNAcylation modification of many proteins, and this modification could be eliminated by OGA, which catalysed the removal of O-GlcNAc^22,23^. Hypoglycemia also increased OGT activity by inducing phosphorylation and attachment to eNOS in an AMPK-dependent manner. This indicated that low glucose increased eNOS O-GlcNAcylation through an additional signaling pathway, independent of the increase of HBP flux and probably distinct from hyperglycemia-induced O-GlcNAcylation previously reported^24^. As a nutrient-sensitive modification site, the increased O-GlcNAcylation of eNOS may be initially triggered by metabolic dysregulation and function in the metabolic memory in endothelial cells^12,13,23^. Rat arteries exposed to high glucose exhibited an increase in eNOS O-GlcNAcylation, which impaired NO-dependent arteriolar dilations^25^. Endothelium-dependent decreased vasodilatation was closely correlated with the decrease of NO^26^. Insulin improved vasodilation by increasing the activity and expression of eNOS^27^. Moreover, hypoglycemia increased vascular blood flow in normal rats whereas decreased endothelium-dependent relaxation in diabetes patients^5,6^. Our results showed that the NO content in low-glucose treated BAECs was higher than that in the control group. After insulin injection, NO content and vasodilation increased in hypoglycemia rats, while no difference in vasodilation was revealed with insulin injection after Compound C treatment compared with the control group. These results demonstrated that low glucose facilitated NO release leading to aortic vasodilation in rats, this way is dependent on AMPK.

Previous investigations revealed the significance of hyperglycemia-induced O-GlcNAcylation of eNOS inhibited the activity of its enzyme by increasing O-GlcNAcylation in eNOS, and phosphorylation of O*-*linked serine at Ser1177 was also decreased^12,13,24^. However, this study did not observe this reciprocal activity when hypoglycemia induced an increase in eNOS O-GlcNAcylation both in vitro and in vivo. The eNOS O-GlcNAcylation site under hypoglycemia condition differed from that of hyperglycemia. We demonstrated that our S1177A-eNOS mutant still underwent O-GlcNAcylation, and that glucose deprivation induced this modification. Based on these factors, we hypothesized that eNOS might contain a potential O-GlcNAcylation site that was modified in response to glucose deprivation. Through HPLC–MS, a novel glucose deprivation-dependent O-GlcNAcylation site was identified in eNOS. And the identities of the novel O-GlcNAcylation site was confirmed by transfecting and expressing site-specific mutant eNOS proteins based on the MS predicted sites. After transfection, HEK293 cells and BAECs were incubated in hypoglycemic conditions, the O-GlcNAcylation of T866A-eNOS was not increased, showing that Thr866 was a novel O-GlcNAcylation site. Moreover, compared with T866A-eNOS, the activity of WT-eNOS was increased 8 to 10 folds. These data suggested that the regulation of eNOS activity under glucose deprivation occurred partly through the regulation of O-GlcNAcylation. Despite this was a pioneering study to construct novel O-GlcNAcylation site modification causing functional alterations in eNOS, these data were somewhat consistent with previous results showing that glucose deprivation increased eNOS activity^5^.

In our experiment, eNOS was first purified from cultured cells by affinity precipitation to ensure that NO synthesized by cell components or tissues other than eNOS was eliminated. Moreover, we excluded another positive regulatory site of eNOS to ensure that eNOS activity was only related to O-GlcNAcylation. Physiological concentrations of insulin were known to increase eNOS activity by increasing phosphorylation at Ser1177^28^. This effect of insulin was eliminated because cells were treated in “pure” glucose deprivation conditions with no eNOS activators. Hence, the only factor that affected the post-translational modification of eNOS was a decrease in glucose concentration. eNOS was closely related to the pathophysiological processes of cardiovascular diseases including hypertension, atherosclerosis, and diabetes^29^. Reduced activity of eNOS was detected in the aorta of diabetic rats^30^. Our results showed that low glucose promoted eNOS O-GlcNAcylation at T866A, which enhanced the activity of eNOS. Therefore, the novel modification reported here may provide a basis for the development of new energy-sensitive targets to prevent obesity-induced angiosclerosis. This target may contribute to preventing the development and progression of severe vasospasm associated with diabetes.

There are also several limitations to this study. As acetylation is another nutrient-sensitive post-translational modification of eNOS, its effect on eNOS activity in hypoglycemic conditions must be clarified in subsequent studies. In the future, effects of T866A eNOS mutant on endothelial cell signaling transduction and its protein–protein interaction should be investigated.

## Conclusions

Our study demonstrated that glucose deprivation increased eNOS O-GlcNAcylation in vivo and in vitro. Glucose deprivation induced O-GlcNAcylation activation, possibly via AMPK-OGT pathway, indicating that Thr866 was a novel O-GlcNAcylation site involved in glucose deprivation-mediated NO synthesis.

## Supplementary information

Supplementary Information

## Data Availability

The datasets used and analyzed during the current study are available from the corresponding author on reasonable request. All data generated or analyzed during this study are included in this published article and its supplementary information files.

## Abbreviations

eNOS: Endothelial nitric oxide synthase
BAECs: Bovine aortic endothelial cells
OGT: O-GlcNAc transferase
AMPK: AMP kinase
NO: Nitric oxide
HPLC-MS: High performance liquid chromatography-mass spectrometry
SiRNA: Small interfering RNA (siRNA)
