# Supplementary material for: Regulation of O-GlcNAcylation on endothelial nitric oxide synthase by glucose deprivation and identification of its O-GlcNAcylation sites
Source: Sci Rep. 2020 Nov 9;10:19364. doi: 10.1038/s41598-020-76340-7 (PMC7652922; doi:10.1038/s41598-020-76340-7)
Supplement: Supplementary file 1 — Supplementary Information [file 41598_2020_76340_MOESM1_ESM.pdf]

Supplementary Information for

Regulation of O-GlcNAcylation on Endothelial Nitric Oxide Synthase by Glucose Deprivation and Identification of its O-GlcNAcylation Sites

An He[1], Shupeng Hu[1], Qiangzhong Pi[1], Yongzheng Guo[1], Yang Long[1], Luo Suxin[1], Yong Xia[1,2,*]
[1]Division of cardiology, The First Affiliated Hospital of Chongqing Medical University, Chongqing 400016, China.
[2]Institute of Life Science, Chongqing Medical University, Chongqing 400016, China.

***Correspondence should be addressed to:** Yong Xia, **Email:** cqmu_cardiology@163.com

Running title: Hypoglycemia activates eNOS via O-GlcNAcylation

Contents

1. Supplementary Figure Legends S1-S16
2. Supplementary Figure S1-S16

Supplementary Figure Legends

Supplementary Figure S1. Full western blot pictures shown in Figure 1A.

Supplementary Figure S2. Full blots for blots shown in Figure 1B.

Supplementary Figure S3. Full blots for blots shown in Figure 1C.

Supplementary Figure S4. Full blots for blots shown in Figure 2A.

Supplementary Figure S5. Full blots for blots shown in Figure 2B.

Supplementary Figure S6. Full blots for blots shown in Figure 2C.

Supplementary Figure S7. Full blots for blots shown in Figure 3B.

Supplementary Figure S8. Full blots for blots shown in Figure 4A.

Supplementary Figure S9. Full blots for blots shown in Figure 4B.

Supplementary Figure S10. Full blots for blots shown in Figure 4C.

Supplementary Figure S11. Full blots for blots shown in Figure 4D and 5D.

Supplementary Figure S12. Full blots for blots shown in Figure 5A.

Supplementary Figure S13. Full blots for blots shown in Figure 5B.

Supplementary Figure S14. Full blots for blots shown in Figure 5C.

Supplementary Figure S15. Full blots for blots shown in Figure 6C.

Supplementary Figure S16. Figures of supplementary experiments to reviewers

Supplementary Figure S17. Full blots for blots shown in Supplementary Figure S16 C

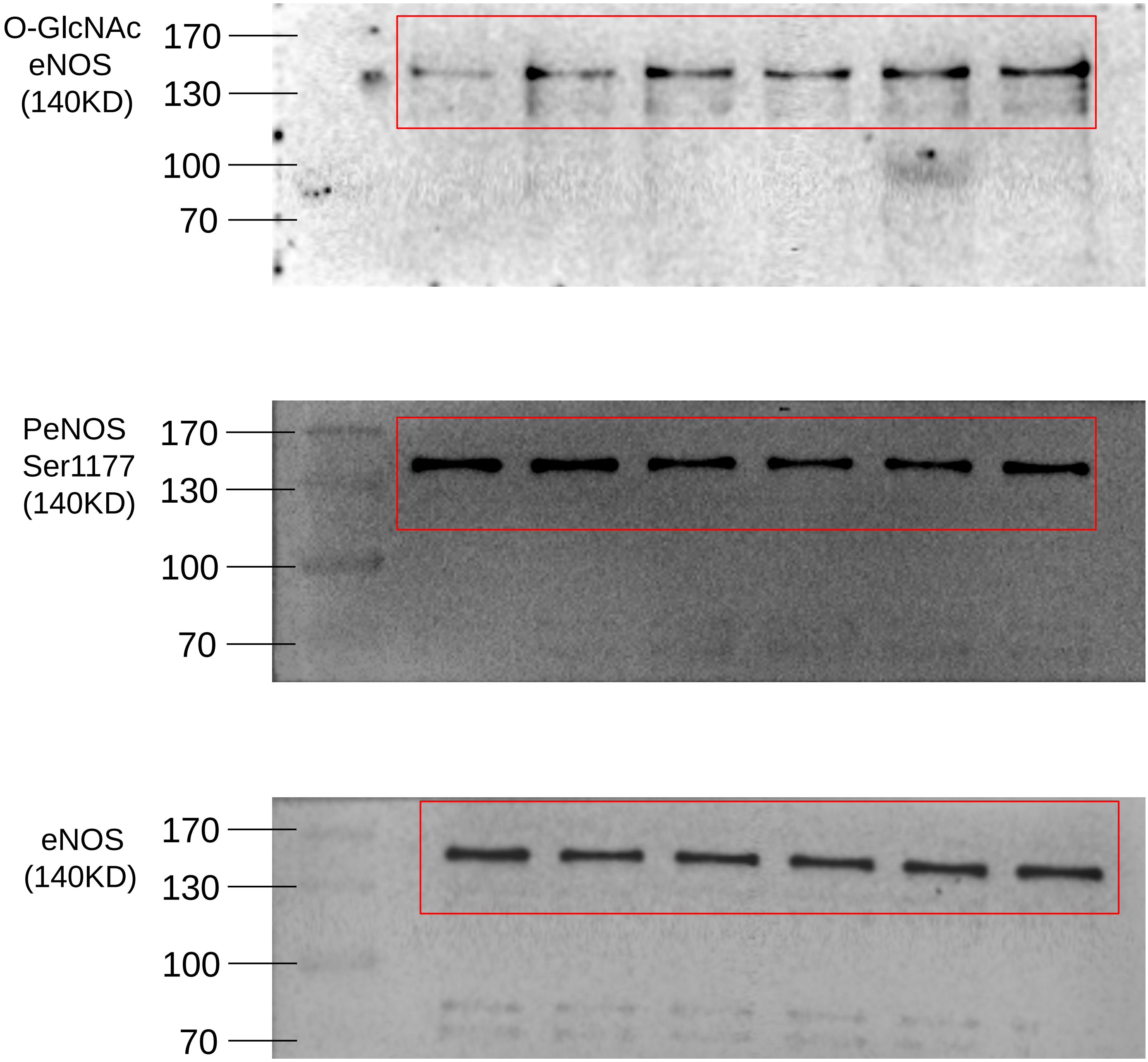

Supplementary Figure S1

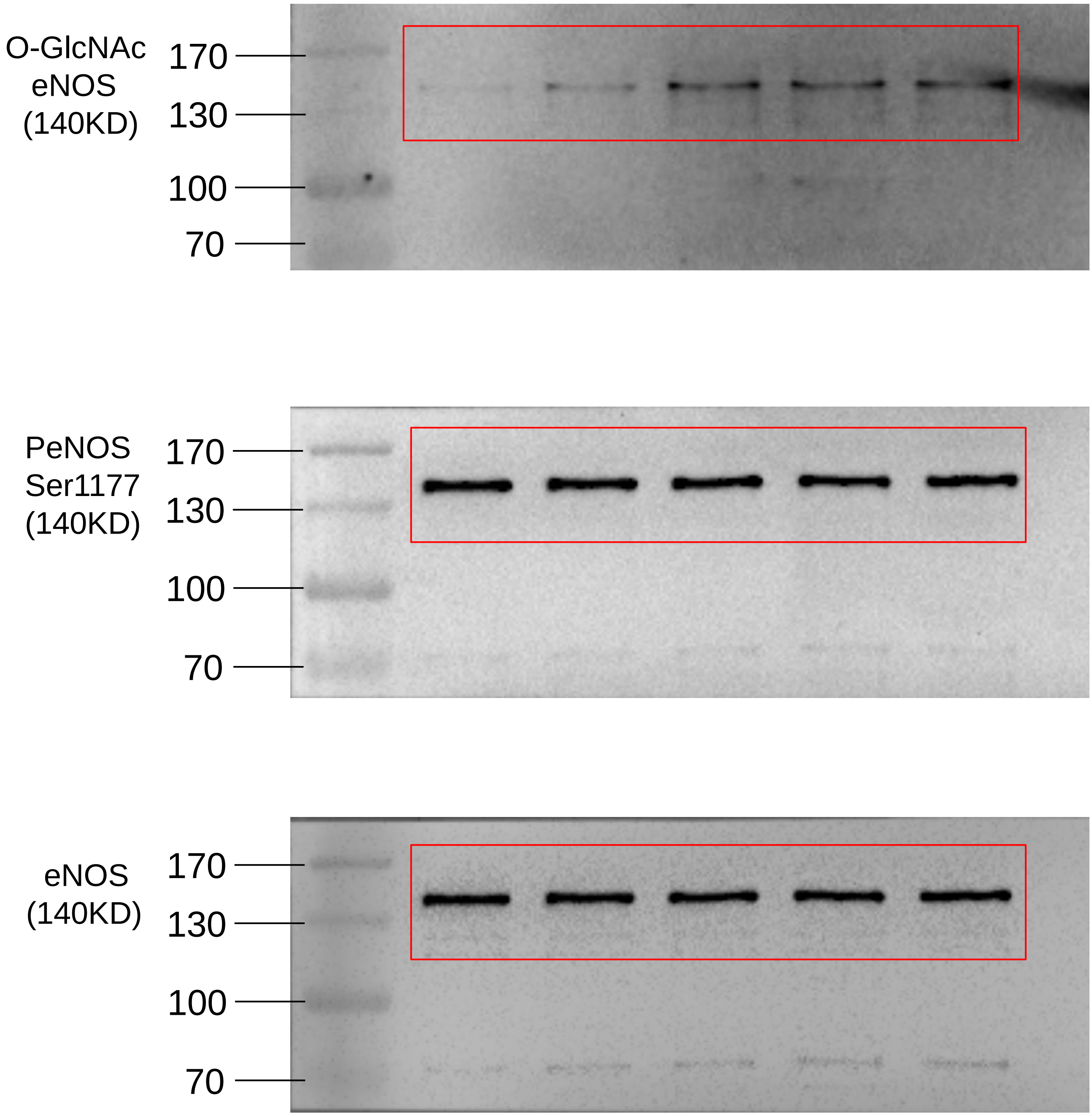

Supplementary Figure S2

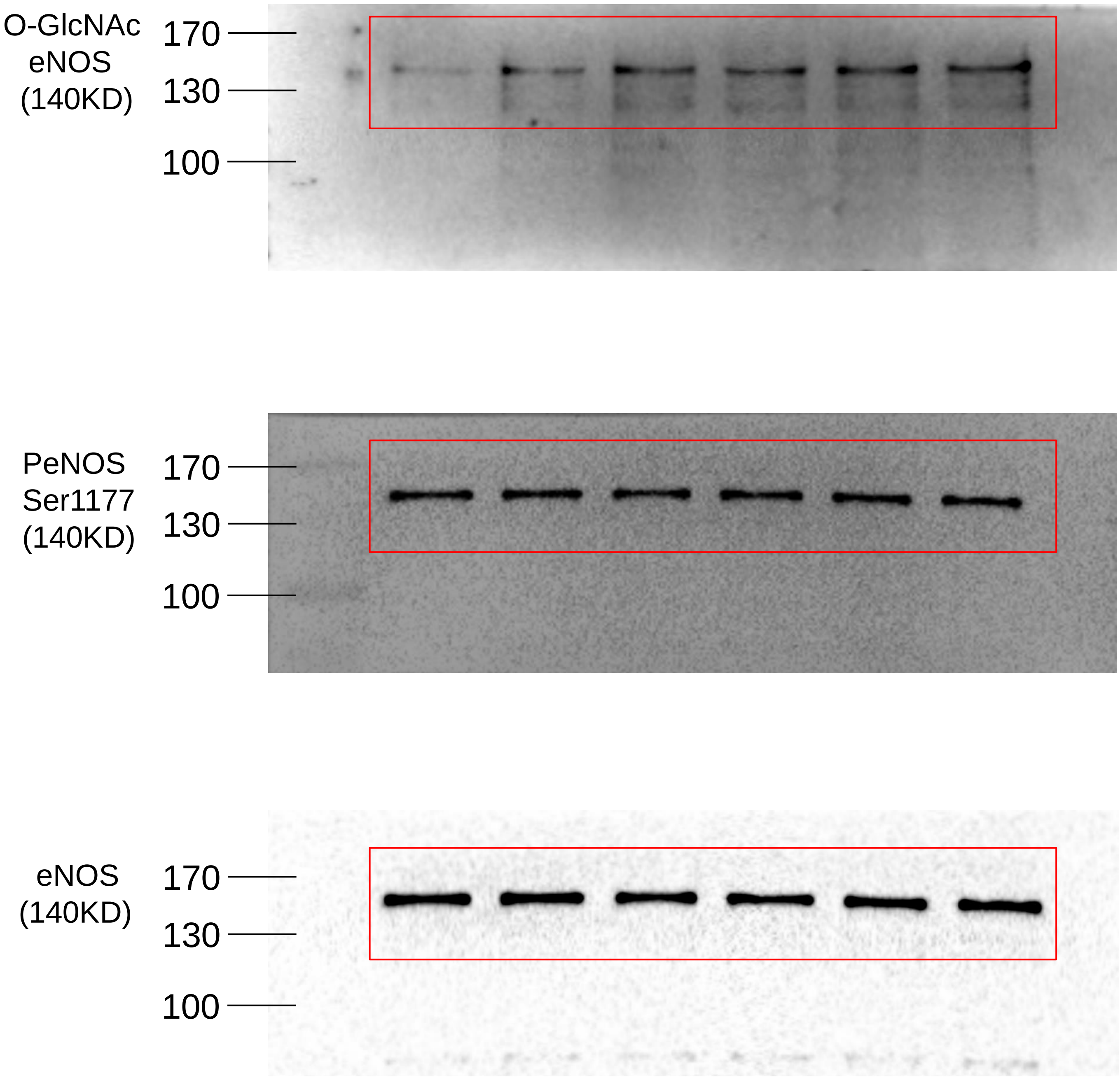

Supplementary Figure S3

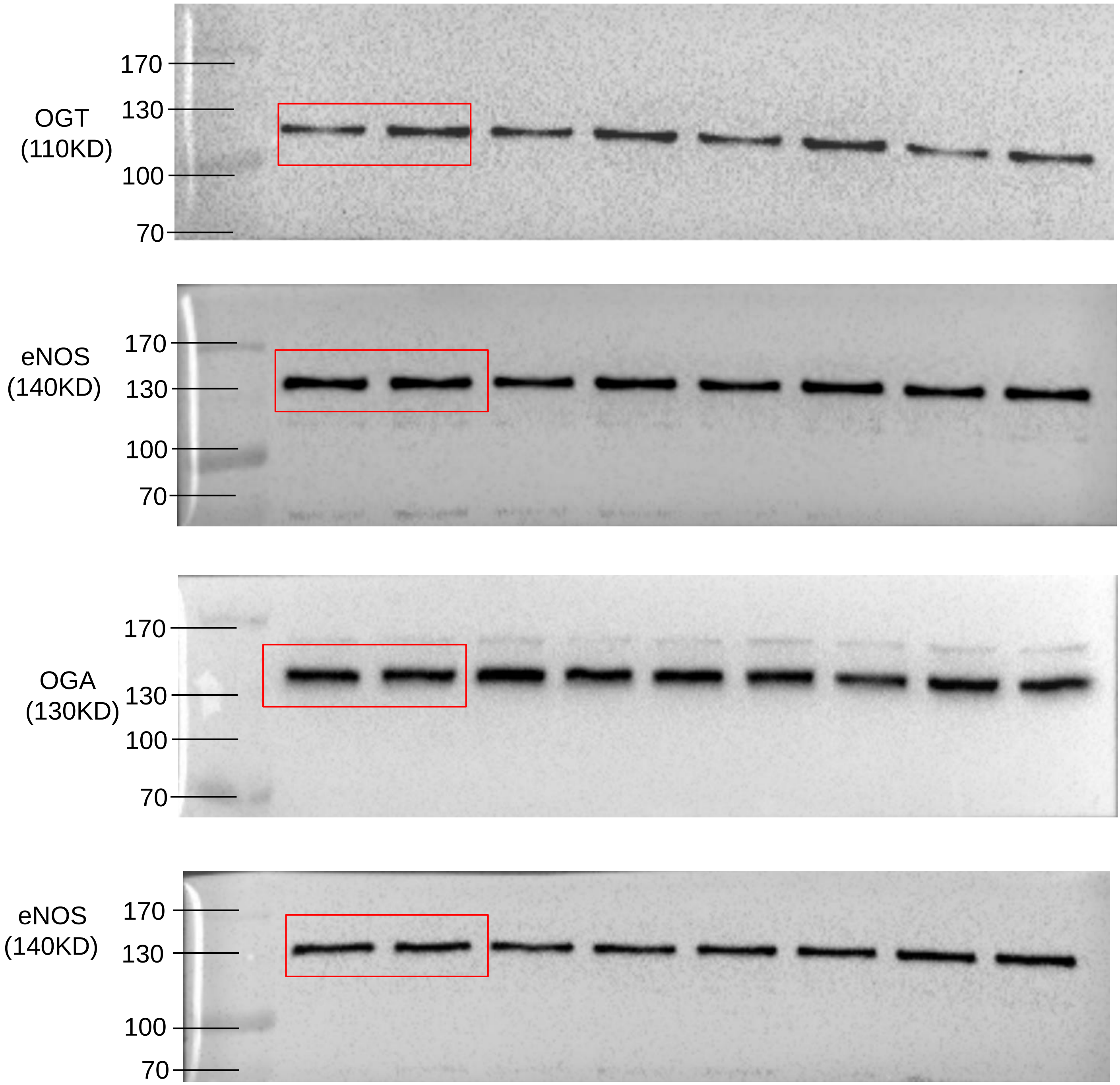

Supplementary Figure S4

The red boxes represent the blots used in the Figure 2A

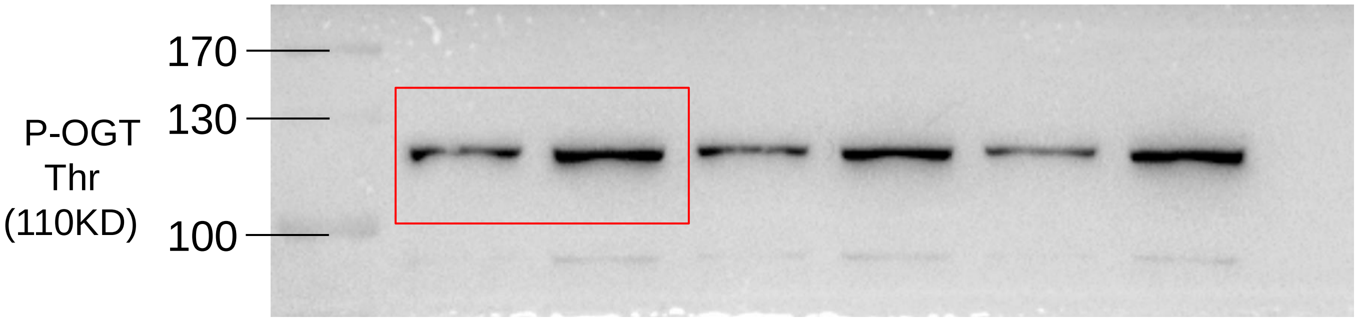

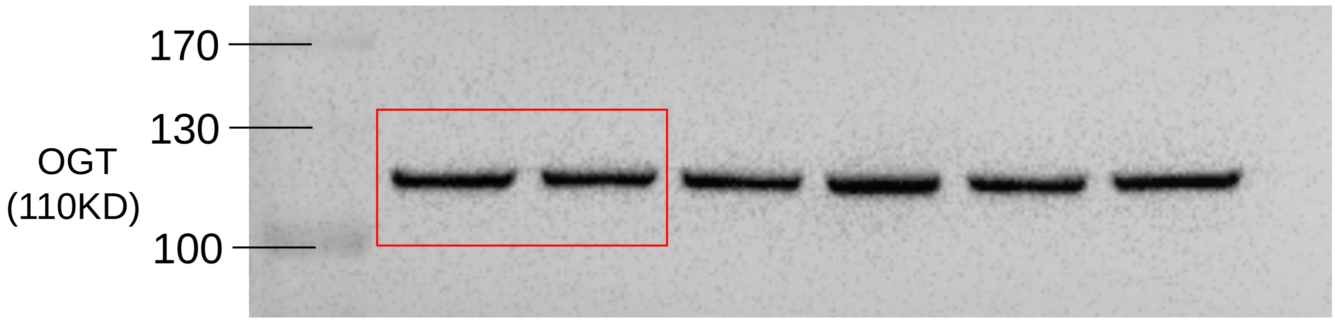

Supplementary Figure S5

The reasons for why the original blots provided are tightly cropped are following: there only exist one target proteins which is purified OGT (down)/P-OGT (up) needs to be determined by immunoblotting. And, the molecular weight of OGT is 110Kda. Hence we only reserve part of PVDF membrane (molecular weight range: 100-130 Kda) for suiting our target proteins. In summary, this is the reason why this original blot is tightly cropped.

O-GlcNAc
eNOS
(140KD)

OGT
(110KD)

eNOS
(140KD)

P-AMPK
Thr172
(63KD)

AMPK alpha1
(63KD)

β-actin
(42KD)

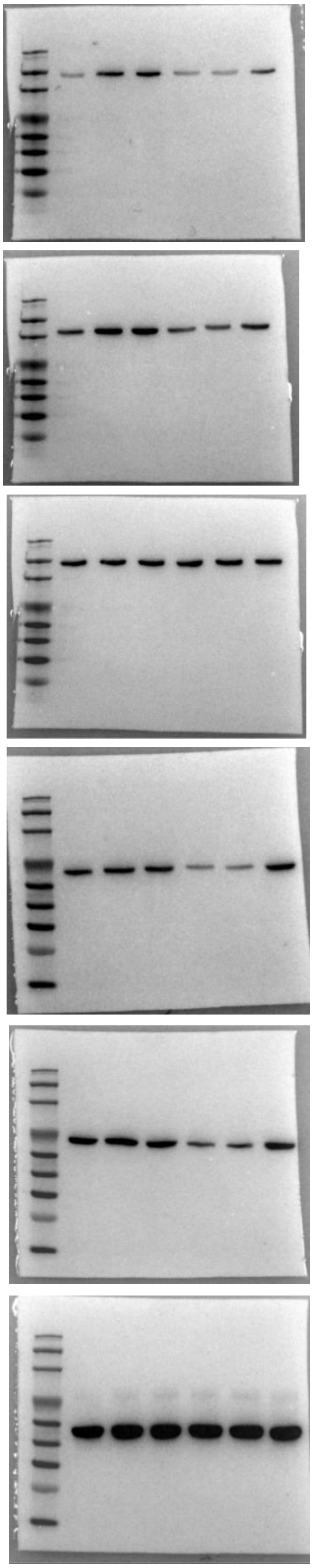

Supplementary Figure S6

Marker is 250/150/100/90/70/50/40/35/25/15 from the top down

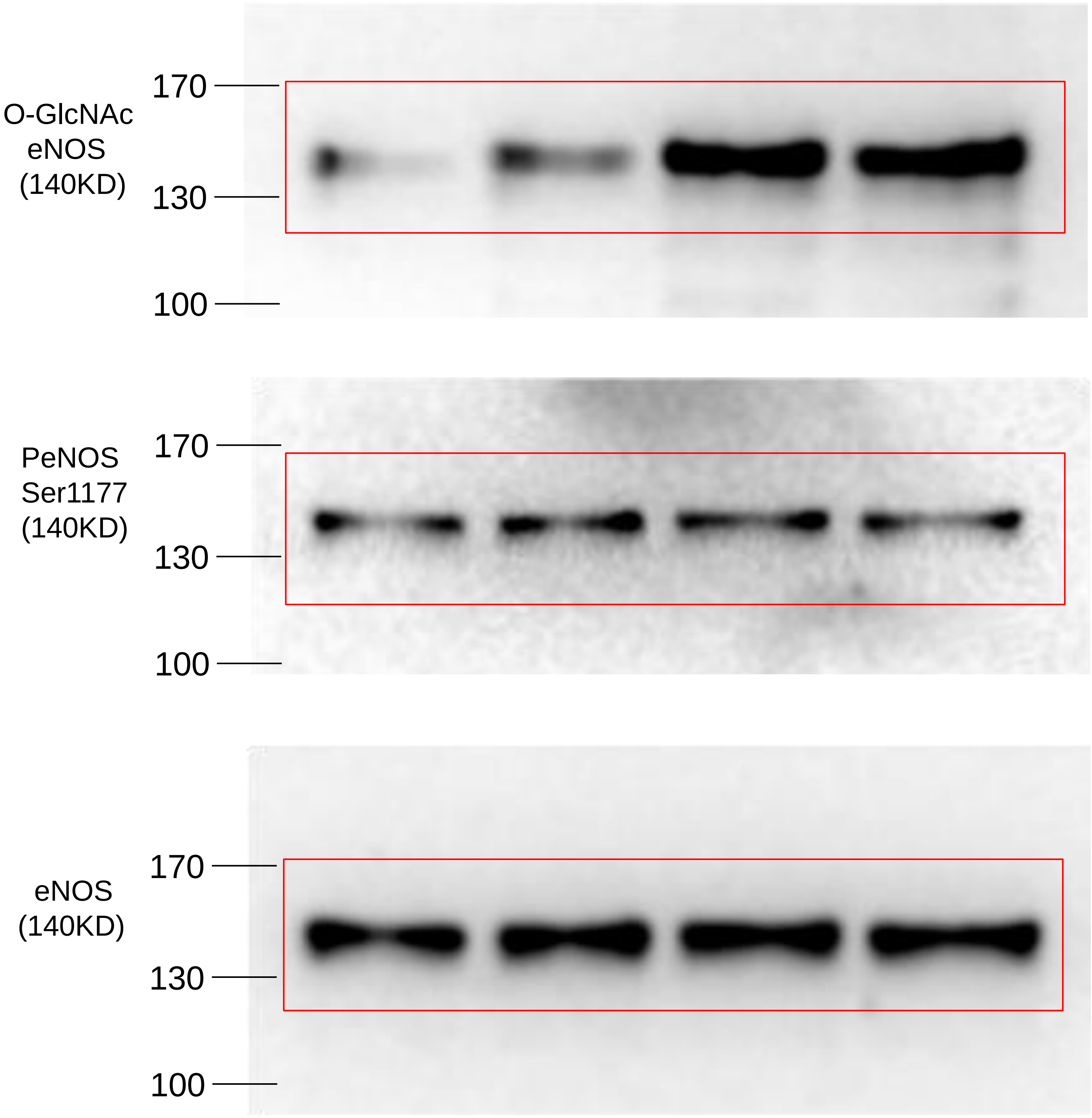

Supplementary Figure S7

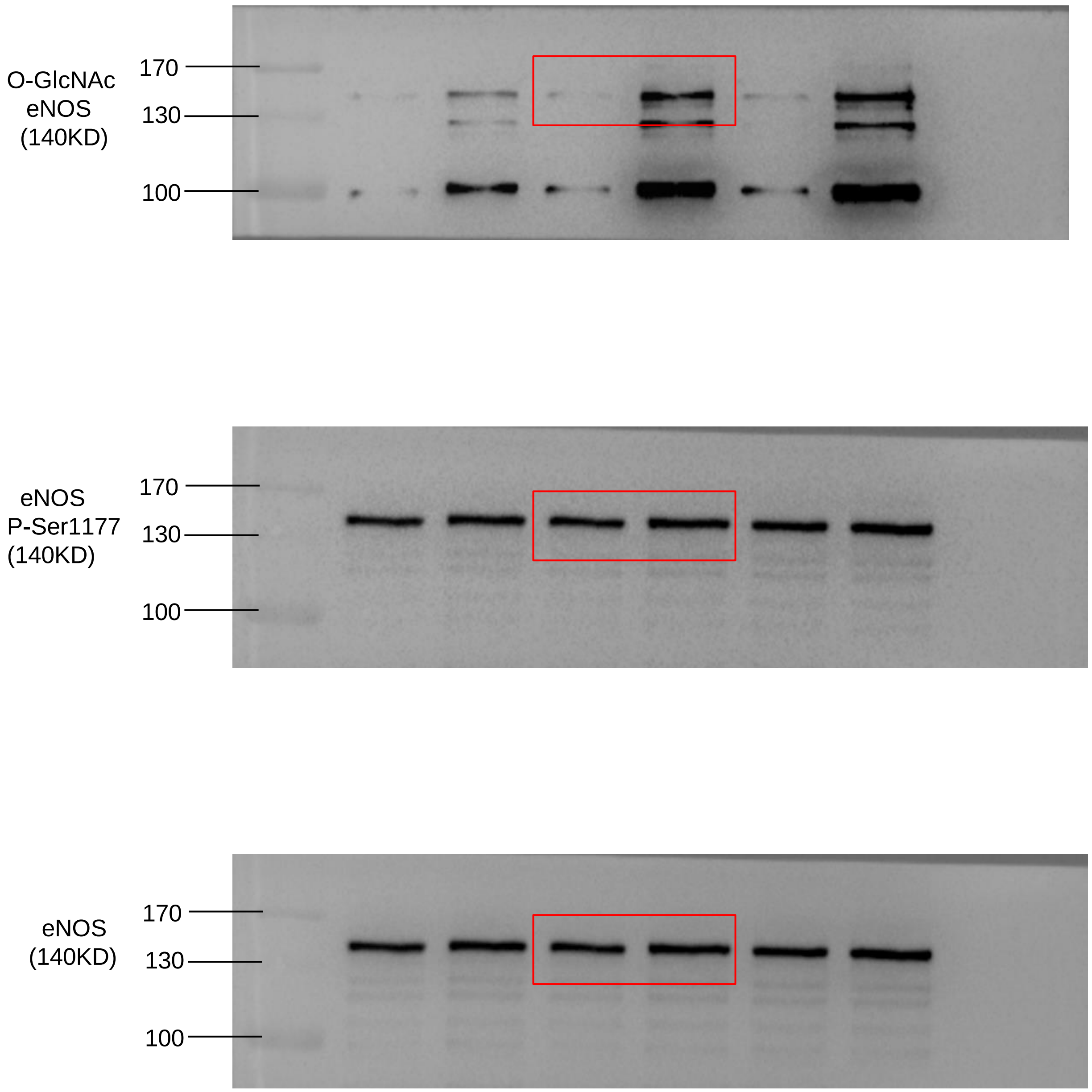

Supplementary Figure S8

The red boxes represent the blots used in the Figure 4A

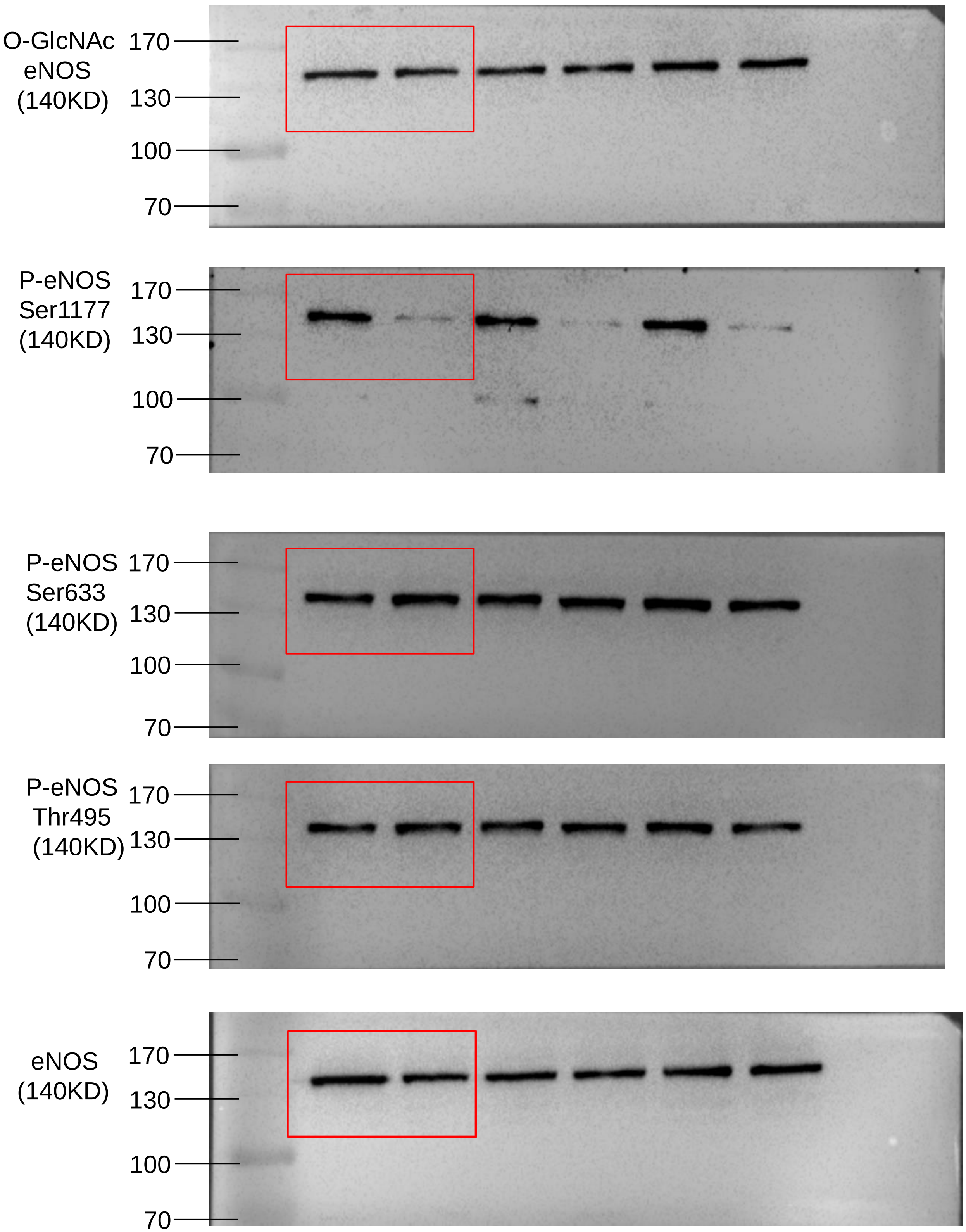

Supplementary Figure S9

The red boxes represent the blots used in the Figure 4B

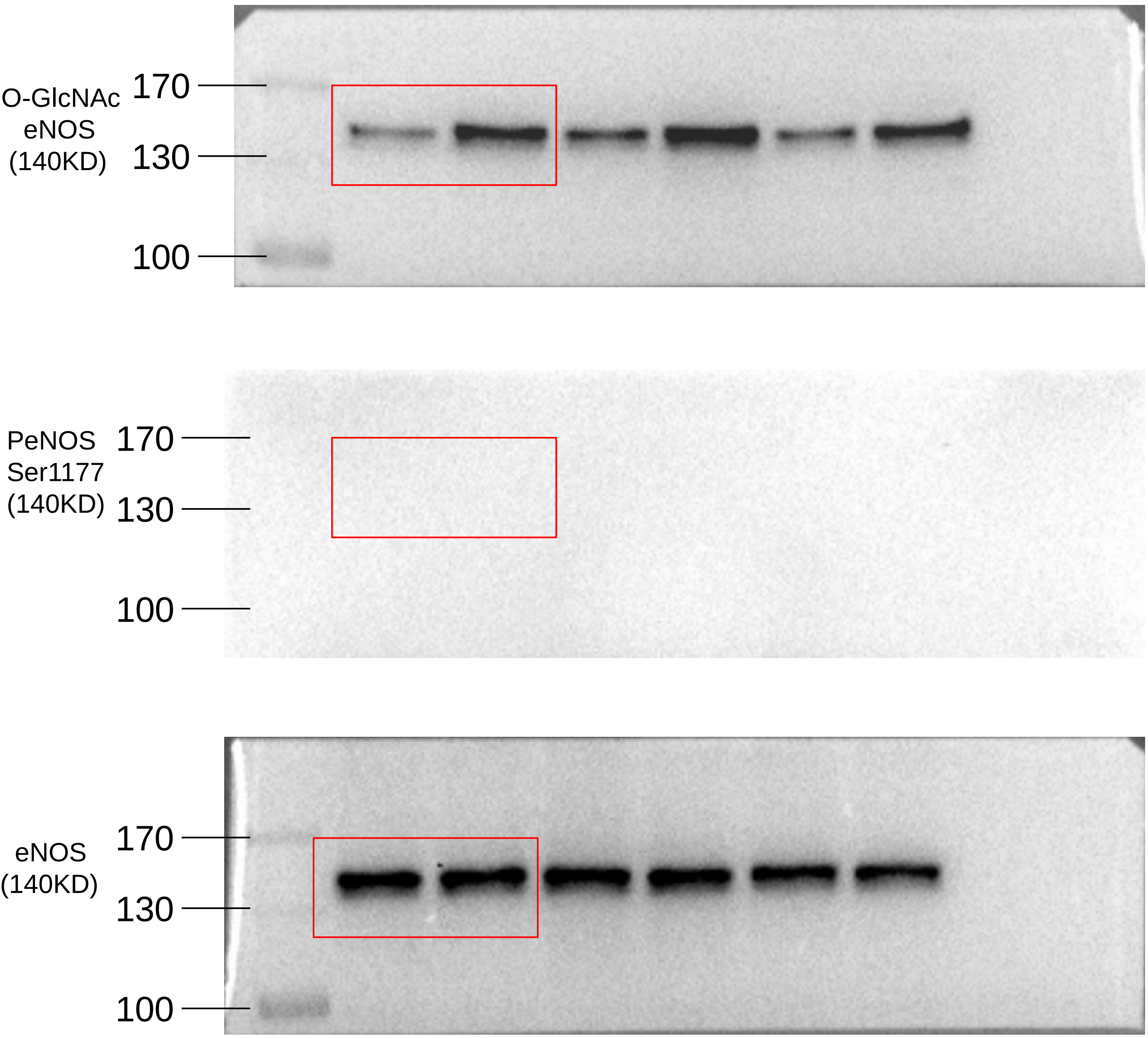

Supplementary Figure S10

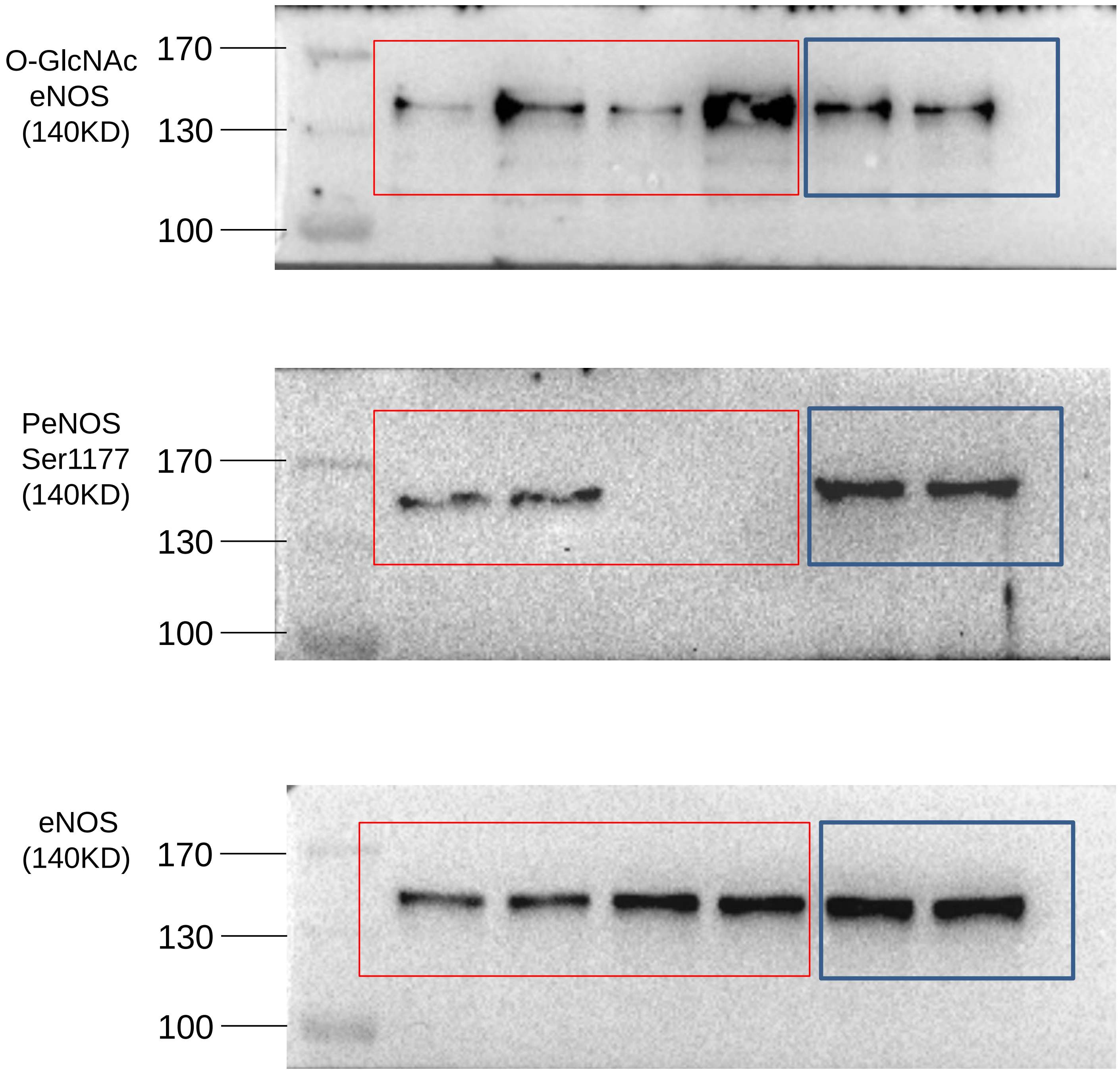

Supplementary Figure S11

The red boxes represent the blots used in the Figure 4D
The blue boxes represent the blots used in the Figure 5D

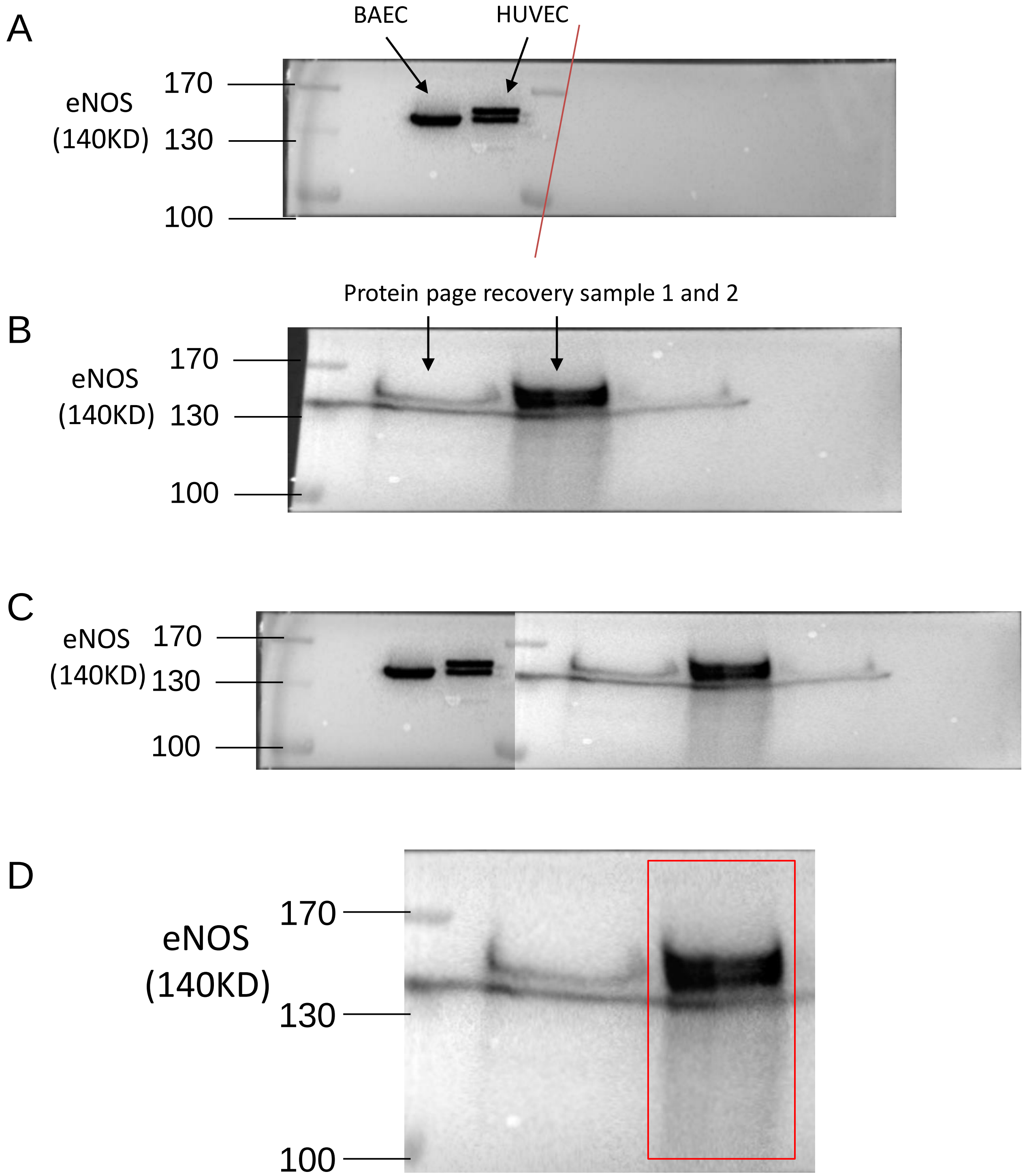

Supplementary Figure S12

(A) and (B) from the same gel. A: The whole membrane was exposed and the internal control signal (left) was strong, results in the inability to see eNOS in B B: Cut the gel in A and re-expose to display eNOS. C: Merge A and B according to marker position. D: Sample 2 was used for HPLC-MS/MS.

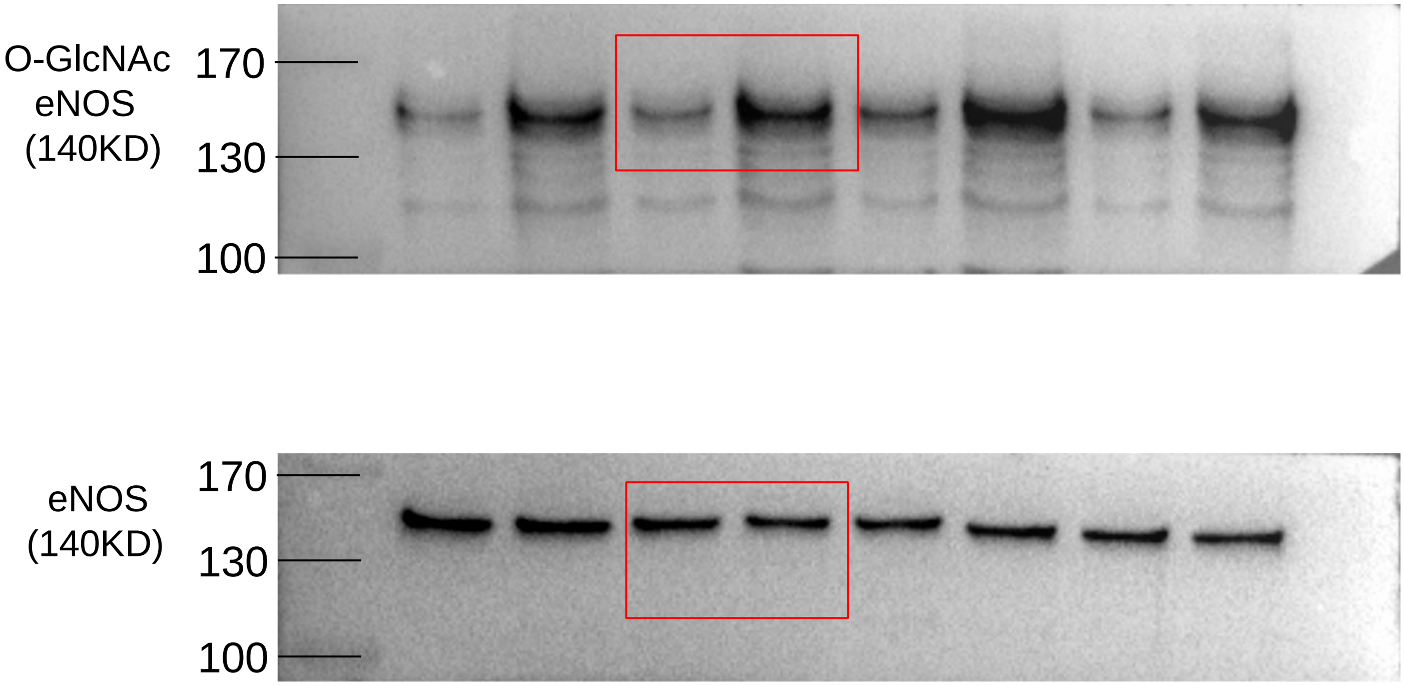

Supplementary Figure S13

The red boxes represent the blots used in the Figure 5B

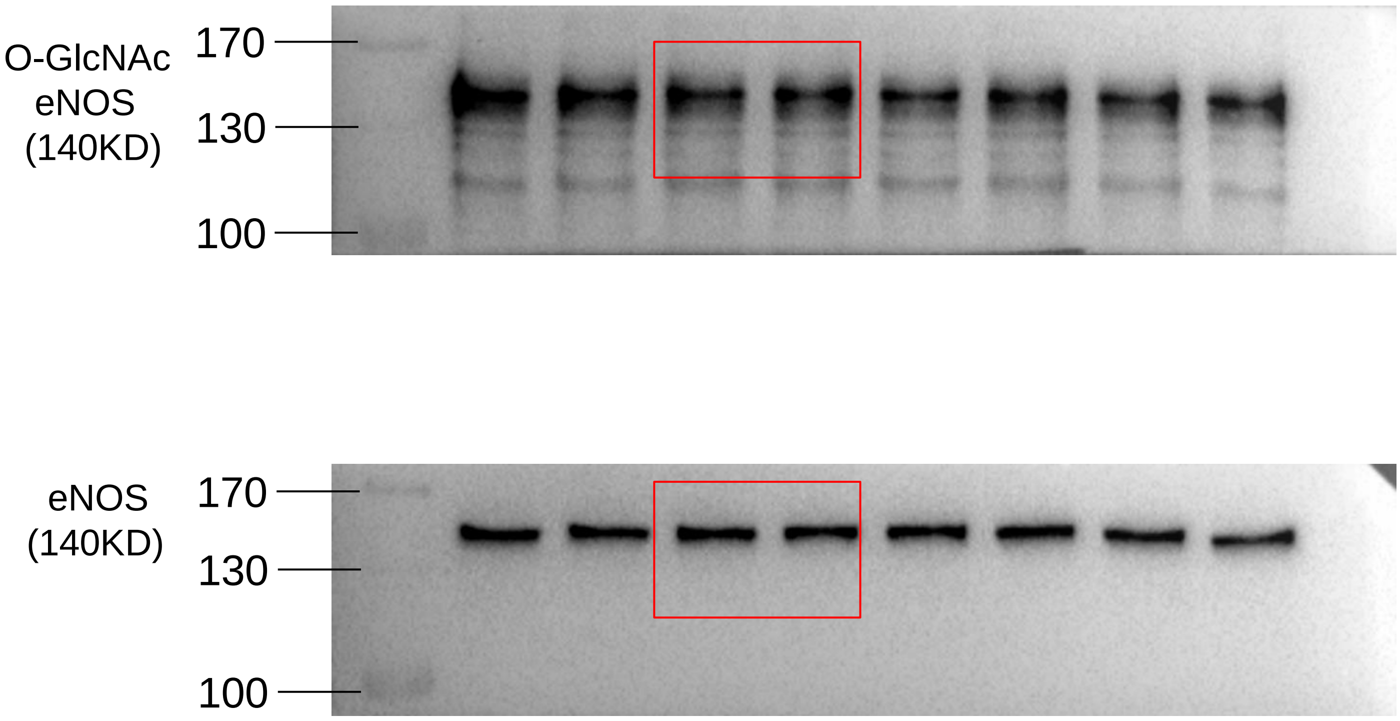

Supplementary Figure S14

The red boxes represent the blots used in the Figure 5C

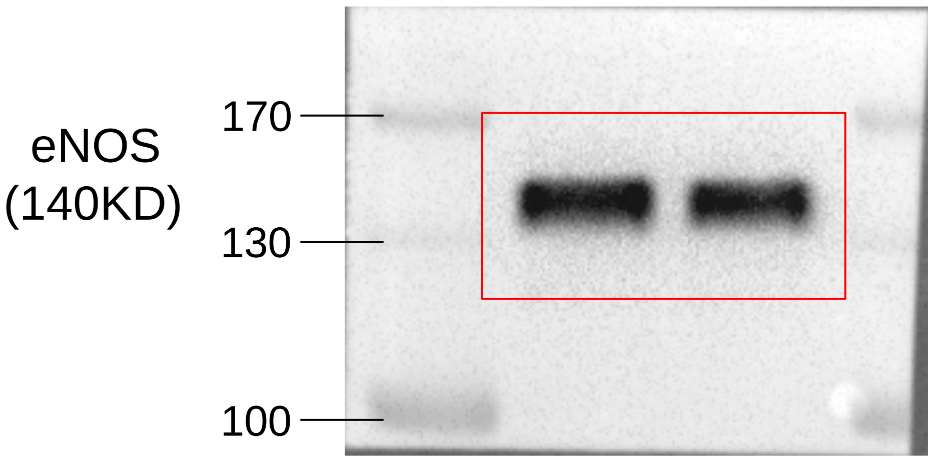

Supplementary Figure S15

There only exist two target proteins (WT-eNOS (left), T866A-eNOS(right)) need to be determined by immunoblotting. And, the molecular weight of eNOS is 140Kda. Hence we only reserve part of PVDF membrane (molecular weight range: 100-170 Kda) for suiting our target proteins. This is the reason why this original blot is tightly cropped.

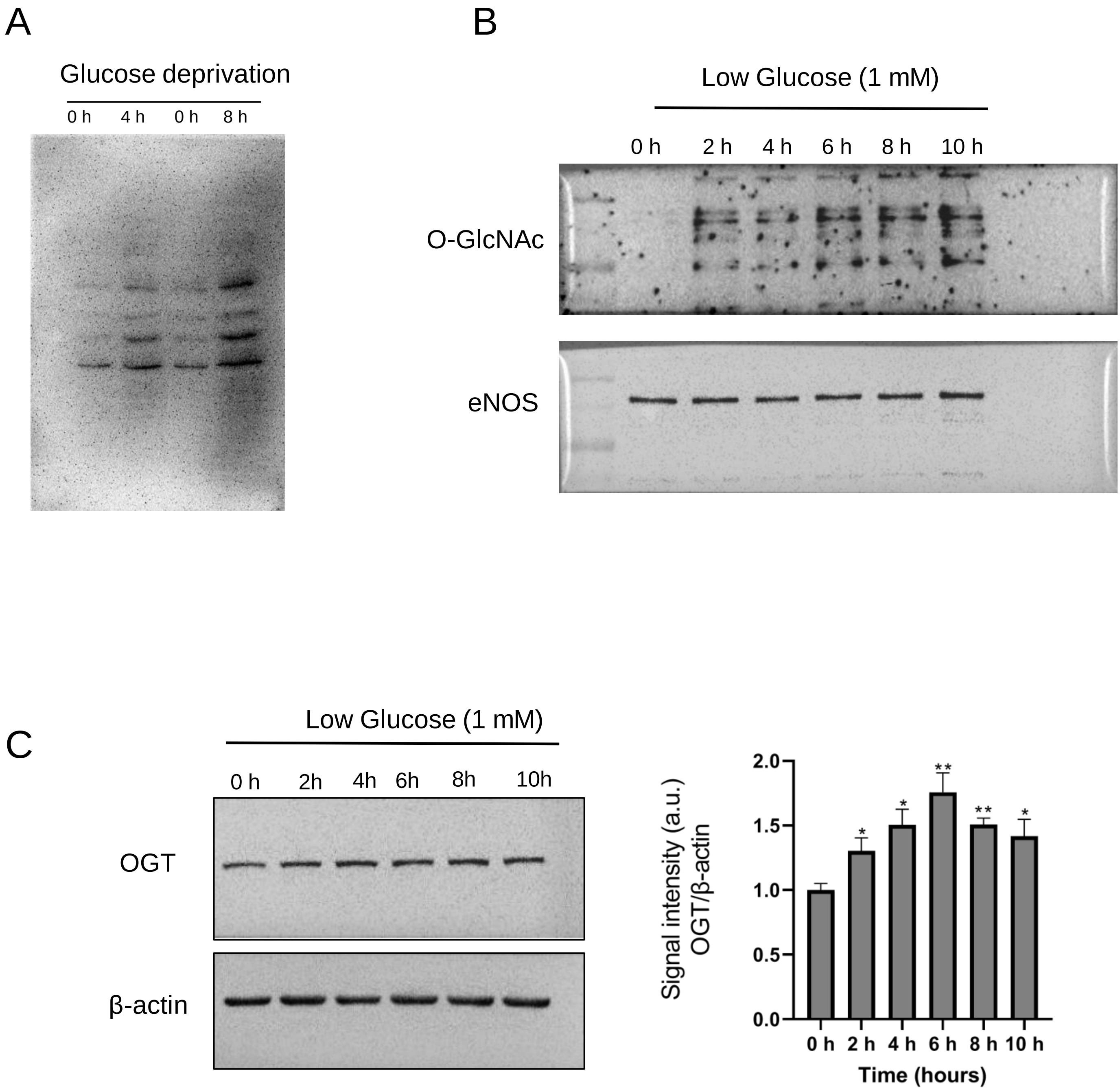

Supplementary Figure S16

Low glucose treatment increased overall O-Glycosylation and OGT levels in BAECs. A, Global O-Glycosylation levels enhanced in glucose deprivon-cultured cells. B, Global O-Glycosylation levels enhanced in low glucose-cultured cells C, OGT expression was upregulated in cells treated with low glucose compared with control (0 h). *, $p < 0.05$; **, $p < 0.01$.

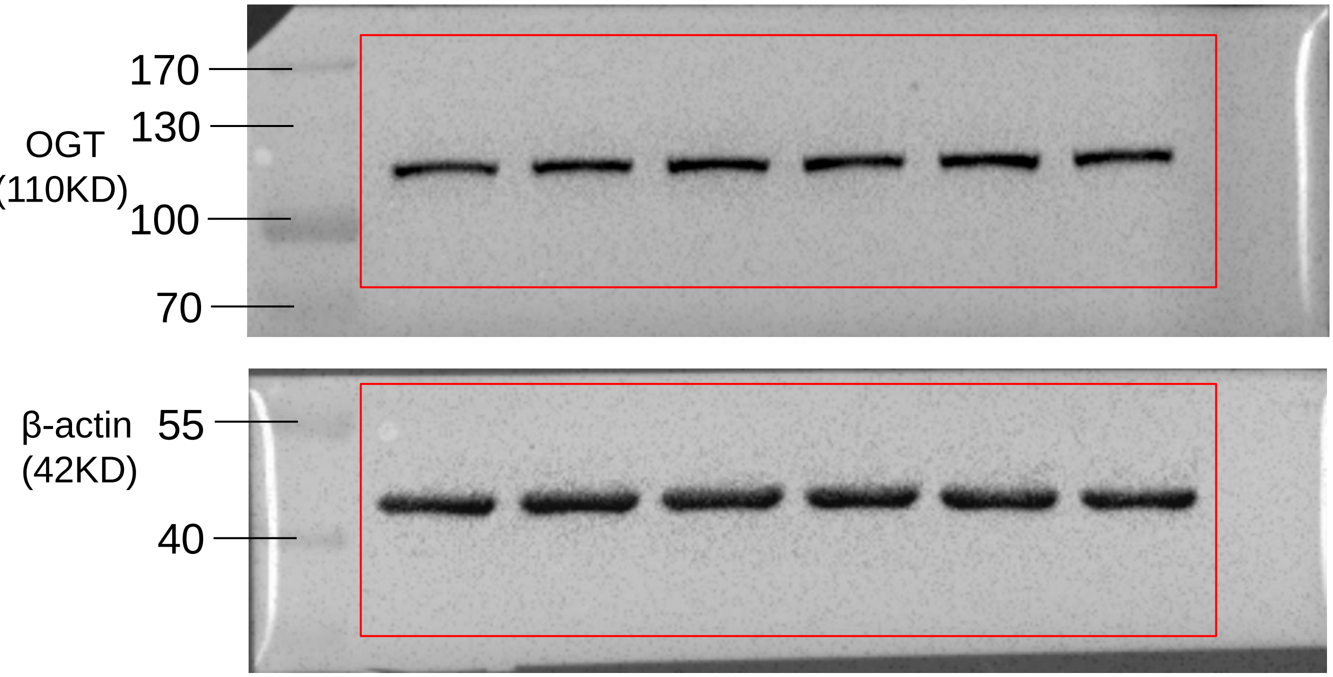

Supplementary Figure S17

The red boxes represent the blots used in the Supplementary Figure S16 C
